# LncRNA MIR31HG fosters stemness malignant features of non-small cell lung cancer via H3K4me1- and H3K27Ace-mediated GLI2 expression

**DOI:** 10.1038/s41388-023-02883-4

**Published:** 2023-11-10

**Authors:** Weiwei Chen, Fei Wang, Xinyuan Yu, Jingjing Qi, Hongliang Dong, Bingjie Cui, Qian Zhang, Yan Wu, Jiajia An, Na Ni, Cuilan Liu, Yuchen Han, Shuo Zhang, Clemens A. Schmitt, Jiong Deng, Yong Yu, Jing Du

**Affiliations:** 1 Medical Research Center, Binzhou Medical University Hospital, Binzhou, 256600 PR China; 2 Department of Oncology, Binzhou Medical University Hospital, Binzhou, 256600 PR China; 3 Department of Hematology and Internal Oncology, Johannes Kepler University Linz, Altenbergerstraße 69, 4040 Linz, Austria; 4 Department of Pathology, Binzhou Medical University Hospital, Binzhou, 256600 PR China; 5 Department of Clinical Laboratory, Binzhou Medical University Hospital, Binzhou, 256603 PR China; 6 Department of Gynecology, Binzhou Medical University Hospital, Binzhou, 256600 PR China; 7 Johannes Kepler University, Altenbergerstraße 69, 4040 Linz, Austria; 8 Kepler University Hospital, Department of Hematology and Oncology, Krankenhausstraße 9, 4020 Linz, Austria; 9 Charité-Universitätsmedizin, corporate member of Freie Universität Berlin, Humboldt-Universität zu Berlin, and Berlin Institute of Health, Medical Department of Hematology, Oncology and Tumor Immunology, and Molekulares Krebsforschungszentrum - MKFZ, Campus Virchow Klinikum, 13353 Berlin, Germany; 10 Max-Delbrück-Center for Molecular Medicine in the Helmholtz Association, Robert-Rössle-Straße 10, 13125 Berlin, Germany; 11 Deutsches Konsortium für Translationale Krebsforschung (German Cancer Consortium), Partner site, Berlin, Germany

**Keywords:** Mechanisms of disease, Non-small-cell lung cancer

## Abstract

Non-coding RNAs are responsible for oncogenesis and the development of stemness features, including multidrug resistance and metastasis, in various cancers. Expression of lncRNA MIR31HG in lung cancer tissues and peripheral sera of lung cancer patients were remarkably higher than that of healthy individuals and indicated a poor prognosis. Functional analysis showed that MIR31HG fosters stemness-associated malignant features of non-small cell lung cancer cells. Further mechanistic investigation revealed that MIR31HG modulated GLI2 expression via WDR5/MLL3/P300 complex-mediated H3K4me and H3K27Ace modification. In vivo MIR31HG repression with an antisense oligonucleotide attenuated tumor growth and distal organ metastasis, whereas MIR31HG promotion remarkably encouraged cellular invasion in lung and liver tissues. Our data suggested that MIR31HG is a potential diagnostic indicator and druggable therapeutic target to facilitate multiple strategic treatments for lung cancer patients.

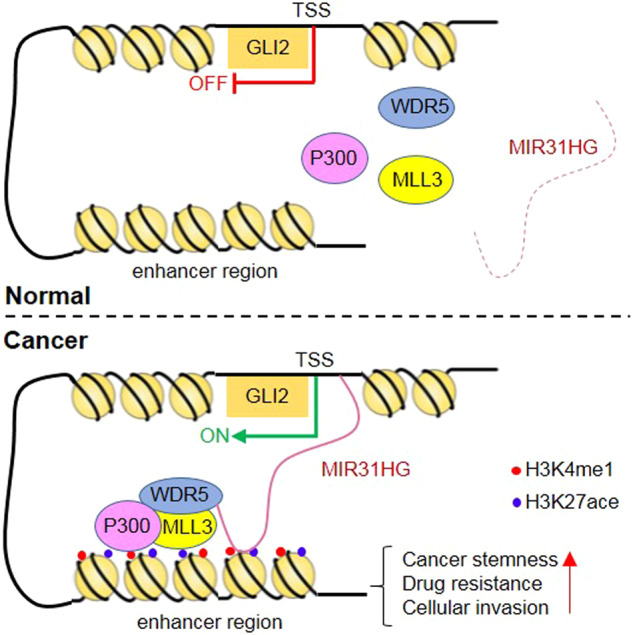

## Introduction

Owing to the increase in atmospheric contamination, lung cancer has become one of the most prevalent malignant diseases worldwide [1]. Based on pathological characteristics, lung cancer is mainly divided into non-small cell lung cancer (NSCLC) and small cell lung cancer (SCLC). NSCLC accounts for approximately 85% lung cancer cases that have benefited from targeted therapy and immunotherapy. However, long-term benefits of the treatment are poor owing to the development of resistance to multiple therapies and distal metastasis at an early stage [2, 3]. Cancer stem-like cells play important roles in tumorigenesis, immune evasion, disease progression, development of multidrug resistance, and cellular invasion capabilities [4, 5]. Therefore, the molecular mechanisms underlying stemness features, including high tumorigenicity, multidrug resistance, distal organ metastasis, and immune evasion, must be identified to develop novel therapeutic targets that can block or reduce malignancy and facilitate NSCLC therapy.

Long non-coding RNAs (lncRNAs), despite comprising only approximately 2% of the human genome, encode proteins that have been discovered to be key regulators of various cellular physiological and pathological processes [6]. lncRNAs are untranslatable RNA transcripts harboring more than 200 nucleotides, whereas microRNAs are 20–24 nucleotide-long, non-coding RNAs. MIR31 has been reported as an oncomir for cigarette smoking-induced human pulmonary carcinogenesis [7]. MIR31HG, the host gene of miR31, is located on human Chr9p21.3 and transcribed in the same orientation as miR31. Although no correlation between miR31 and MIR31HG expression was found in lung cancer specimens, MIR31HG significantly promoted lung adenocarcinoma cell proliferation [8]. Findings on MIR31HG expression in tumor tissues and its roles in malignant disease development are controversial. MIR31HG expression is downregulated in bladder cancer, suggesting a tumor suppressor role in malignant transformation [9]. In pancreatic ductal adenocarcinoma and colorectal cancer, however, MIR31HG expression is elevated, serving as an unfavorable prognostic marker and exhibiting oncogenic features [10, 11].

The Hedgehog cascade, a conventional pathway of stem cell self-renewal and proliferation, is closely related to carcinogenesis and cancer progression [12]. The cascade begins when a signal is terminally transported from plasma to regulate the family of glioma-associated oncogenes (GLIs), which comprise three components, GLI1, GLI2, and GLI3. GLI2 functions as the active transcription factor to initiate downstream signaling molecules (SOX2, ABCG2, and Snail) and induce tumorigenesis, drug resistance, and metastasis [13–15]. Previous studies, including the present study, have demonstrated the essential role of the Hedgehog cascade, particularly its executor GLI1, in cancer stem cell-mediated NSCLC development and progression [16–18]. The Hedgehog cascade can be stimulated in a canonical way, dependent on the translocation of its upstream regulator SMO, or in an uncanonical way, dependent on the expression of aberrant non-coding RNAs [19, 20].

lncRNAs function at the epigenetic, transcription, and post-transcription levels in either a *trans*- or *cis*-manner [21]. For epigenetic modification, lncRNAs may act as protein scaffolds, modulating gene transcription by guiding chromatin-modifying complexes to form active (H3K4me1/2/3 and H3K27Ace) or repressive (H3K9me3 and H3K27me3) histone modifications, which play key roles in switching on and off the gene transcription under pathological conditions such as cancer [21, 22]. Methylation and acetylation at various sites of histone are excreted by histone methyltransferases (such as methyltransferase mixed-lineage leukemia proteins [MLLs]) and acetyltransferases, including P300, respectively [23, 24]. WD repeat domain 5 (WDR5) has been reported to serve as a bridge protein to assist mono-, di-, and tri-methylation in H3K4, initiating cancer stem-like properties such as drug resistance and metastasis of lung cancer cells [25]. An RNA-binding pocket exists on WDR5 for lncRNA or mRNA binding and is necessary for the WDR5-MLL complex to maintain an active chromatin state of target genes in embryonic stem cells [26]. Accumulating data have demonstrated that MIR31HG modulates downstream target genes at the transcriptional level [27, 28]; however, whether MIR31HG modulates the development of multiple therapy-resistance mechanisms, metastasis, and/or immune evasion in NSCLC via histone epigenetic modification remains unknown.

Here, we aimed to investigate how MIR31HG influenced lung cancer stem cell-mediated drug resistance, cellular invasion, and stemness features by epigenetically modulating GLI2 expression using mechanistic and functional analyses. Our findings may help in the development of new prognostic and therapeutic strategies for patients with lung cancer.

## Results

### MIR31HG is overexpressed in tumor tissues and inversely related to clinic prognosis

**Fig. 1:**
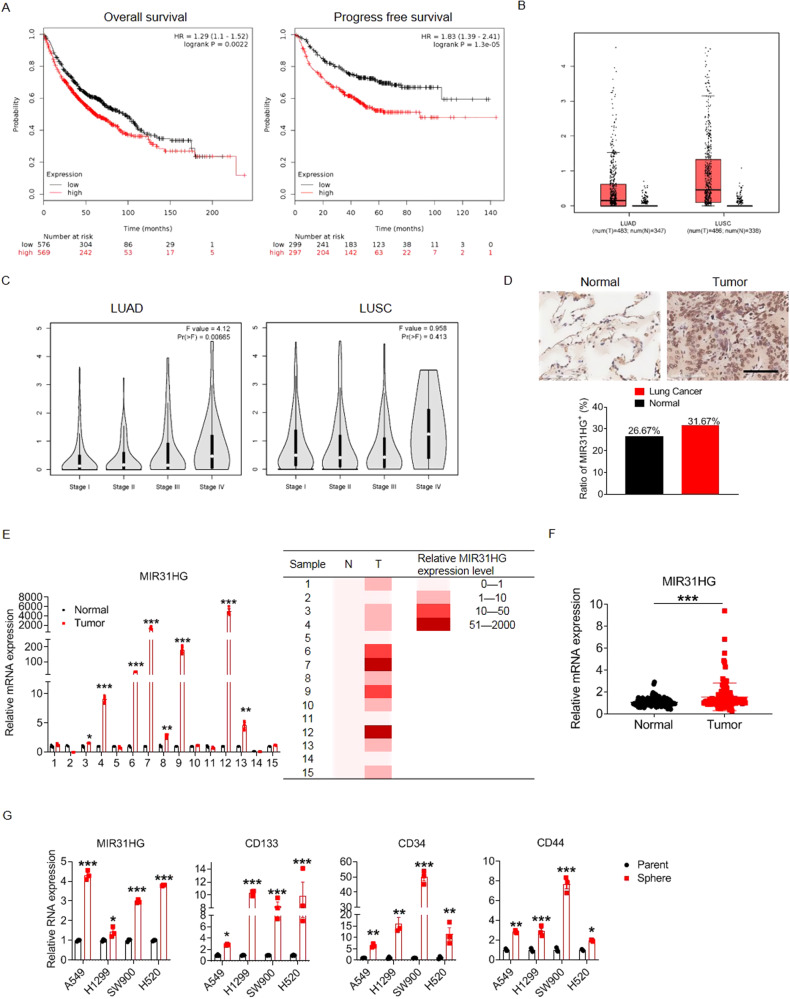
MIR31HG is overexpressed in tumor tissues and inversely related to the clinical prognosis of NSCLC patients. **A** Kaplan–Meier survival analysis of overall and progress-free survival in NSCLC patients according to lncRNA MIR31HG expression. NSCLC non-small cell lung cancer. **B** MIR31HG expression in LUAD and LUSC tumor tissues and normal tissues extracted from the GEPIA database. LUAD lung adenocarcinoma, LUSC lung squamous cell carcinoma. **C** MIR31HG expression in NSCLC tumor tissues from patients at different clinical stages plotted by the GEPIA database. **D** In situ hybridization staining of MIR31HG in 60 paired NSCLC tissues and adjacent non-cancerous tissues (upper panel). Scale bar, 100 µm. Quantification of MIR31HG expression levels between cancer and adjacent non-cancerous tissues (lower panel). **E** MIR31HG transcription levels in 15 paired NSCLC tissues and adjacent non-cancerous tissues detected by RT-PCR. **F** MIR31HG transcription levels in the peripheral serum of 114 healthy populations and 115 lung cancer patients measured by RT-PCR. All of samples were normalized to the healthy 1# peripheral serum and the comparison of significant differences was performed by grouped student’s *t*-test. **G** MIR31HG, CD34, CD44, and CD133 transcription levels in NSCLC parental and stem cell spheroids detected by RT-qPCR. Data are presented as mean ± SEM; significance abundance: *p < 0.05, **p < 0.01, ***p < 0.001.

To explore the correlation between MIR31HG expression and NSCLC prognosis, we drew Kaplan–Meier curves of overall survival (OS) and progress-free survival (PFS) based on data from the Kaplan–Meier Plotter website. Patients with higher MIR31HG expression exhibited poorer prognosis based on OS and PFS analyses (Fig. 1A). MIR31HG was aberrantly overexpressed in both lung adenocarcinoma (LUAD) and lung squamous cell carcinoma (LUSC) tumor tissues compared to levels in normal specimens based on data from the GEPIA database (Fig. 1B). Moreover, MIR31HG was more highly expressed in stage IV lung cancer than in stages I-III, though the difference was not significant (Fig. 1C). Tissue microarray analysis of 60 paired tissues revealed that MIR31HG was more highly expressed in cancerous specimens than in adjacent paired tissues (Fig. 1D). Furthermore, MIR31HG expression was significantly higher in 8 out of 15 lung cancer specimens (53%) than in paired normal tissues (Fig. 1E). Furthermore, the transcription level of MIR31HG in blood serum was higher in patients with lung cancer than in healthy controls (Fig. 1F). Since cancer stem cells account for multidrug resistance of lung cancer, we cultured spheroids from two LUAD (A549 and H1299) and two LUSC (H520 and SW900) cell lines. Quantitative polymerase chain reaction (qPCR) revealed that the transcription level of MIR31HG, as well as three stem cell specific markers (CD133, CD34, and CD44) were significantly higher in cancer stem cell types than in parental cells (Fig. 1G). For functional verification, we take H1299 and H520 cell lines as representation of LUAD and LUSC respectively in following experiments.

### MIR31HG positively regulates stemness-related malignant features in NSCLC cells

**Fig. 2:**
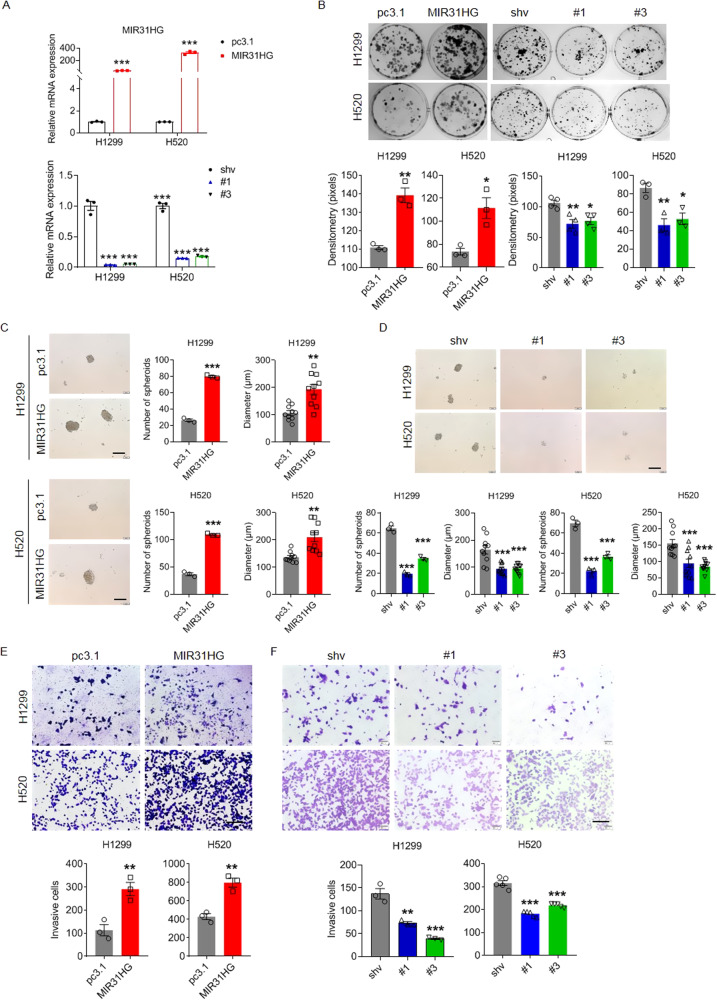
MIR31HG positively regulate stemness-related features in NSCLC cells. **A** Transcriptional level of MIR31HG upon overexpression and shRNA knockdown respectively, as detected by RT-qPCR. pc3.1: pcDNA3.1; MIR31HG: pcDNA3.1 based MIR31HG-overexpressing plasmid; shv: pLV-EGFP vector backbone; #1 and #3: two pLV-EGFP vectors based shRNAs against MIR31HG. **B** Clonal formation assay (upper) and quantification (lower) of NSCLC cells upon forced MIR31HG overexpression or repression. **C** Spheroid formation assay (left) and quantification (right) of NSCLC cells upon exogenous MIR31HG overexpression. Scale bar, 200 µm. **D** Spheroid formation assay (upper) and quantification (lower) of NSCLC cells upon MIR31HG repression. Scale bar, 200 µm. **E**, **F** Invasive capability (upper) and quantification (lower) of NSCLC cells upon MIR31HG overexpression or repression, as detected by transwell staining and histogram quantification. Scale bar, 200 µm. Data are presented as mean ± SEM; significance abundance: *p < 0.05, **p < 0.01, ***p < 0.001.

To explore the functionality of MIR31HG in NSCLC cells, we used MIR31HG overexpression plasmid and shRNA constructs to perform gain- and loss-of-function analysis. qPCR analysis confirmed that MIR31HG was potently overexpressed or repressed when the MIR31HG overexpression plasmid or shRNA constructs, respectively, were introduced via exogenous plasmid transfection in H1299 and H520 NSCLC cell lines (Fig. 2A). Colony formation assay and quantification demonstrated that enforced MIR31HG expression promoted NSCLC cell proliferation, whereas MIR31HG knockdown repressed cell growth (Fig. 2B). Moreover, the spheroid formation assay showed that exogenous MIR31HG stimulated lung cancer stem cell proliferation and knockdown prevent cell expansion (Fig. 2C, D). Transwell assays revealed that exogenous MIR31HG expression remarkably promoted cellular invasion and metastasis of NSCLC cells (Fig. 2E). When silenced with shRNA, invasiveness of NSCLC cells was significantly inhibited compared to that of empty vehicle-transfected cells (Fig. 2F). Would healing assay and quantification validated the pro-invasiveness capability of MIR31HG in NSCLC cells (Fig. S1A, B).

To check the drug sensitivity of NSCLC cells upon MIR31HG genetic manipulation, we tested the IC50 of two EGFR-targeting inhibitors (gefitinib and osimertinib) and two conventional chemotherapy drugs (DDP and GEM). The CCK8 assay revealed that MIR31HG overexpression promoted multidrug resistance, whereas MIR31HG disruption enhanced drug sensitivity against the four tested drugs (Fig. S2A, B). The IC50 and resistance index of individual drugs under MIR31HG up-or downregulation are summarized in tables S5 and S6.

### MIR31HG knockdown represses Hedgehog cascade and downstream stemness markers

**Fig. 3:**
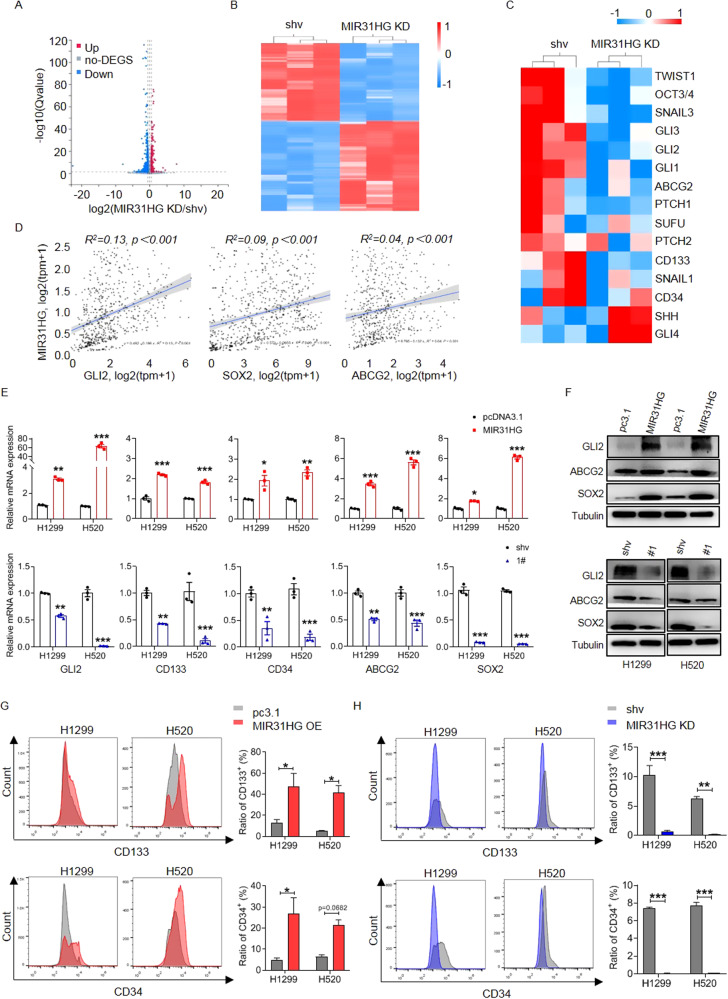
MIR31HG modulates stemness-related genes expression in NSCLC cells. **A** Volcano plot of MIR31HG comparing MIR31HG shv and MIR31HG KD in H1299 cells. **B** Heatmap showing differentially expressed genes between MIR31HG shv and MIR31HG KD in H1299 cells. **C** Heatmap showed enriched expression of stemness and Hedgehog related genes in MIR31HG KD group based on the RNA-seq analysis. Color bar indicates gene expression in scale. **D** Positive correlation between GLI2, SOX2, ABCG2 expression, and MIR31HG expression by generated from TCGA database. **E**, **F** The mRNA and protein levels of GLI2, CD133, CD34, ABCG2, and SOX2 upon MIR31HG overexpression or knockdown compared to levels in empty vector detected by RT-qPCR and immunoblotting. **G**, **H** The expression of cancer stemness markers CD133 and CD34 were detected by flow cytometry upon MIR31HG overexpression or knockdown compared to levels in empty vector. Data are presented as mean ± SEM; significance abundance: *p < 0.05, **p < 0.01, ***p < 0.001.

For mechanistic investigation, RNA Sequencing was performed with H1299 cells to screen candidate signaling cascade. Compared to control cells transfected with shRNA empty vector, 2139 and 1861 genes were up- and downregulated correspondingly in MIR31HG silenced cells (Fig. 3A, B). Since our previous study demonstrated that the Hedgehog cascade is related to lung cancer stemness features and multidrug resistance, we focused on whether MIR31HG functions via the Hedgehog pathway. Heatmap clearly demonstrated that components of Hedgehog cascade and downstream stemness markers were significantly downregulated in MIR31HG knockdown cells (Fig. 3C). Since GLI1 and GLI2 are two terminal key transcription factors modulating the Hedgehog cascade, we take GLI2 which is more potently downregulated upon MIR31HG and its downstream stemness-related genes for further investigation. Correlation analysis based on TCGA database indicated that expression level of MIR31HG were positively related to the level of GLI2, SOX2, and ABCG2 (Fig. 3D). qRT-PCR assay validated that GLI2, CD133, CD34, ABCG2, and SOX2 were significantly upregulated by enforced MIR31HG expression and downregulated upon MIR31HG knockdown (Fig. 3E). Immunoblot verified that MIR31HG positively modulated protein level of GLI2, ABCG2, and SOX2 (Fig. 3F). Furthermore, stemness markers CD133 and CD34 were detected by flow cytometry and immunofluorescence. Expression of CD133 and CD34 were significantly increased in NSCLC cells by exogenous MIR31HG (Fig. 3G and Fig. S3). Accordingly, expression of CD133 and CD34 were remarkably decreased in NSCLC cells by upon MIR31HG disruption (Fig. 3H and Fig. S3)

To validate the genetic and functional specificity of MIR31HG in lung cancer cells, MIR31HG shRNA was single transfected or double transfected with MIR31HG overexpression plasmid into H520 cells. qRT-PCR assay revealed that exogenous MIR31HG expression rescued downregulated transcription of GLI2, SOX2, ABCG2, CD133, and CD44 (Fig. S4A). Phenotypic assay demonstrated that MIR31HG overexpression counteracted MIR31HG knockdown mediated anti-proliferative and anti-metastasis effect (Fig. S4B, C). In addition, MIR31HG overexpression offsetted inhibition of spheroid formation exerted by MIR31HG shRNA in H520 cells (Fig. S4D).

### MIR31HG modulates the GLI2 transcription via WDR5/MLL/P300 complex-mediated H3K4me1/H3K27ace modification

**Fig. 4:**
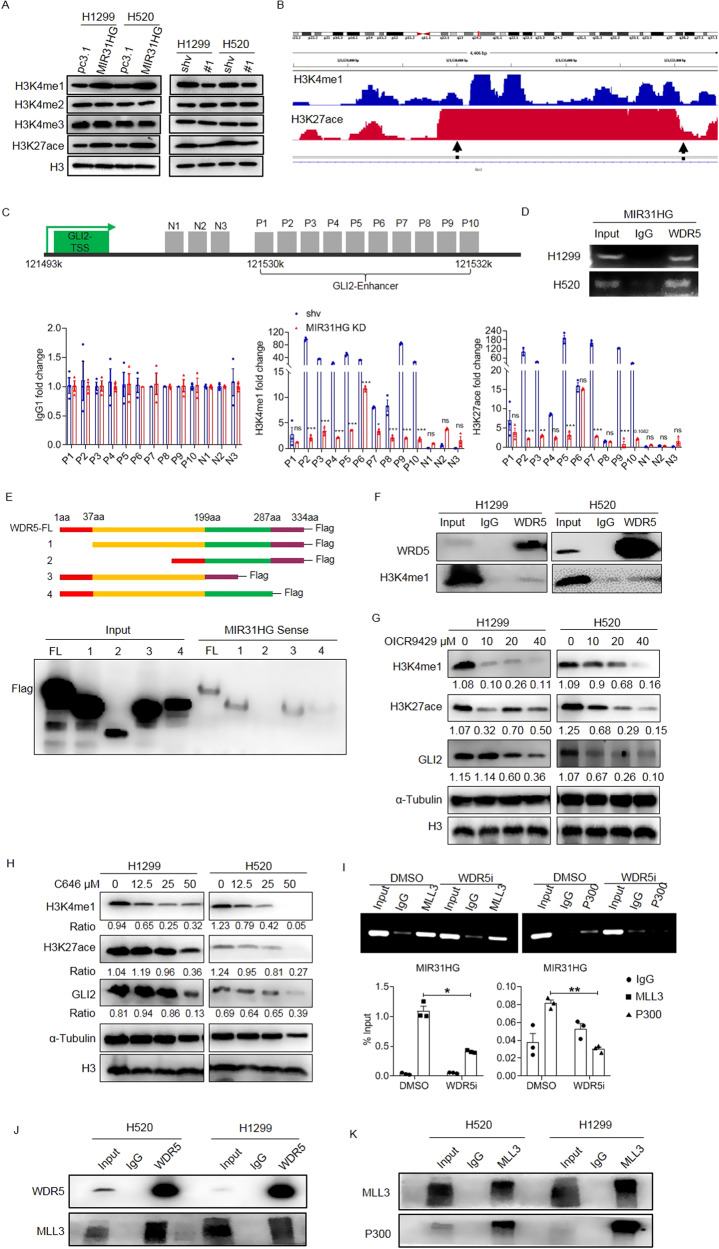
MIR31HG modulates GLI2 expression via WDR5/MLL3/P300 complex-mediated H3K4me1 and H3K27ace modification in NSCLC cells. **A** Expression level of H3K4me1/2/3 and H3K27Ace in NSCLC cells upon MIR31HG overexpression or knockdown detected by immunoblot. H3 was used as an internal control for nuclear protein. **B** CUT&Tag sequencing of H1299 genome precipitated by H3K4me1 or H3K27ace antibody zoomed in at the GLI2 enhancer area in H1299 cells. **C** CUT&Tag assay followed by RT-qPCR of the H3K4me1 + /H3K27ace+ region of GLI2 enhancer by H3K4me1 or H3K27ace antibody, or IgG as a control. The primers P1-P10 were designed for the GLI2 enhancer region, and N1-N3 locate in front of P1-P10 region where no peaks detected as negative control. **D** Amplification of MIR31HG transcripts upon WDR5 antibody precipitation, as detected by the RIP assay. IgG antibody-precipitated specimens were used as negative control. Whole cell lysate was labeled as the input and used as the positive control. **E** Schematic diagram (upper panel) showing Flag-tagged full-length WDR5 protein or its truncates and interaction between biotin-sense labeled MIR31HG and peptides detected by RNA pulldown assay (lower panel). **F** Interaction between WDR5 and H3K4me1 modification detected by co-IP immunoblotting. **G**, **H** Protein levels of H3K4me1, H3K27ace, and GLI2 in NSCLC cells followed by WDR5 (**G**) or P300 (**H**) inhibitor administration. α-Tubulin and H3 were used as internal controls for whole cell and nuclear protein, separately. **I** RT-PCR (up) and RT-qPCR (down) of MIR31HG following RIP assay precipitated by MLL3, P300, and IgG as the negative control. DMSO: solvent of WDR5 inhibitor; WDR5i WDR5 inhibitor applied at 20 µM. **J** Interaction between WDR5 and MLL3 detected by co-IP immunoblot in H520 and H1299 cells. **K** Interaction between MLL3 and P300 detected by co-IP immunoblot in NSCLC cells. Data are presented as mean ± SEM; significance abundance: * *p* < 0.05, ** *p* < 0.01, *** *p* < 0.001.

Bioinformatic analysis with UCSC genome browser indicated multiple sites modified by H3K4me1 and H3K27ace on enhancer and promoter region of GLI2 (Fig. S5A). We assumed that MIR31HG may play a pivotal role in the epigenetic modification of GLI2 regulatory element. Immunoblot staining revealed that MIR31HG modulated the overall level of H3K27ace and H3K4me1 modification across the genome but did not modify overall H3K4me2 or H3K4me3 levels in H1299 and H520 cells (Fig. 4A). Cleavage under targets and tagmentation (CUT&Tag) sequencing of H1299 cells also showed H3K4me1 and H3K27ace modifications at multiple sites across Chr2 (q14.2), where the GLI2 gene is located (Fig. S5B). Sequences from 121,530 to 121,532 kb, which was predicted to be the enhancer area of GLI2 by the UCSC database, were H3K4me1- and H3K27ace-positive (Fig. 4B). Ten pairs of primers covering the above area were designed, and qPCR showed H3K4me1 and H3K27ace modifications in control H1299 cells (blue bars in Fig. 4C). Both H3K4me1 and H3K27ace modifications in the enhancer area of GLI2 were reduced when MIR31HG was knocked down in H1299 cells (Fig. 4C).

Next, we explored how MIR31HG modulates the modification of H3K4me1 and H3K27ace. The RNA immunoprecipitation (RIP) assay showed that MIR31HG directly combined with WDR5, a scaffold protein for H3K4 methylation modification (Fig. 4D). RNA pulldown followed by immunoblot corroborated the binding between MIR31HG sense strand and WDR5 (Fig. S5C). catRAPID omics database predicted three potential binding domains of MIR31HG in WDR5, PF00400 (35–287aa), PF08662 (81–139aa) and PF12894 (74–197aa). Based on the predicted binding area, full length and four truncated peptides of WDR5 tagged with Flag were constructed for RNA pulldown assay. Immunoblot displayed clearly that the peptide 2 which lack amino acid from 37 to 199 of WDR5 did not bind with MIR31HG (Fig. 4E). The protein co-immunoprecipitation (co-IP) assay revealed that H3K4me directly interacted with WDR5, further suggesting that MIR31HG may guide the WDR5 complex to the GLI2 enhancer region for H3K4 mono-methylation (Fig. 4F). Because the WDR5/MLL3 complex and P300 have been reported to account for H3K4me1 and H3K27ace, respectively [29, 30], we used inhibitors targeting WDR5, P300, and MLL1 as controls to analyze their influence on GLI2 expression. Pharmacological inhibition of both WDR5 and P300 decreased protein levels of GLI2, H3K4me1, and H3K27ace in a dose-dependent manner based on immunoblot results (Fig. 4G, H). MLL1 inhibitor, on the contrary, had only neglectable influence on protein levels of GLI2 and H3K4me (Fig. S5D). RIP analysis revealed that WDR5 inhibitor significantly (*p* < 0.01) decreased MIR31HG enrichment upon MLL3 or P300 precipitation in H1299 cells (Fig. 4I). Immunoblot assay demonstrated that WDR5 inhibitor do not influence the level of MLL3 and P300 themselves therefore exclude the possibility that decreased MLL3 or P300 protein produced false positive (Fig. S5E). To detect the interaction among components, co-IP data showed binding of WDR5/MLL3 and MLL3/P300 in NSCLC cells (Fig. 4J, K). Furthermore, compared to shRNA empty transfected control cells, MIR31HG knockdown significantly decreased interaction between WDR5/MLL3, WDR5/P300, and WDR5/H3K4me (Fig. S5F). MIR31HG knockdown not only decreased stability of the WDR5/MLL3/P300 complex but also reduced binding capability of the complex with GLI2 enhancer area (Fig. S5F, G). These data suggested that MIR31HG guided the WDR5/MLL3/P300 complex via WDR5 to form a H3K4me1 + /H3K27ace+ active modification region in the regulatory area of GLI2.

To further explore weather MIR31HG has specific binding sites on GLI2 gene for histone epigenetic modification, we performed target prediction of MIR31HG on GLI2 genomic region with strict parameter settings (Nt = 80 and offset = 30) (reference https://www.nature.com/articles/s41596-018-0115-5) and found several potential binding sites. One of them located at 121477830 to 121477909, about 15 kb upstream of TSS which locate at 121493000 of GLI2 gene. One of the binding sites locates between 121537204 to121537280, about 5 kb away from the H3K4me/H3K27ace modification site validated in Fig. 4B. This suggested that MIR31HG might on one hand guide the WDR5/MLL3/P300 complex for histone modification in the enhancer area of GLI2, and one the other hand form a loop with promoter region to facilitate GLI2 transcription.

### MIR31HG promotes stemness-related features of lung cancer cells via positive regulation of GLI2

**Fig. 5:**
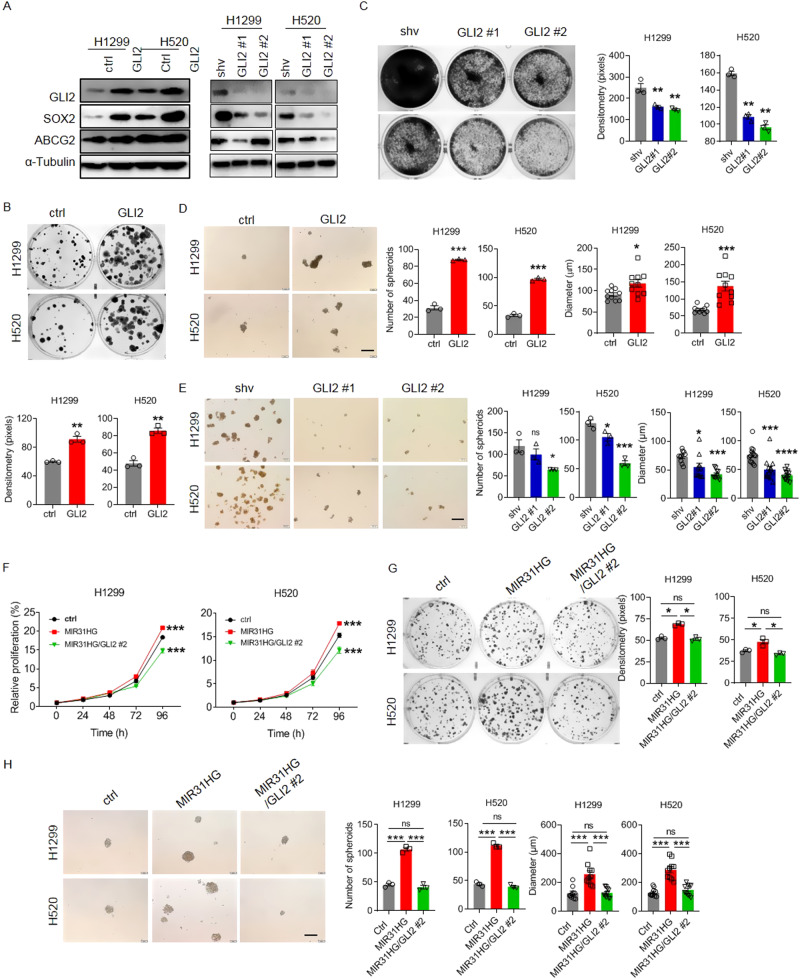
GLI2 acts as a downstream target of MIR31HG and promotes stemness features of NSCLC cells. **A** Expression of GLI2, SOX2, and ABCG2 in NSCLC cells upon transfection of GLI2 overexpressing or knockdown plasmid respectively checked by immunoblot. **B** Clonal formation assay (up) and quantification (down) of NSCLC cells following GLI2 overexpression. **C** Clonal formation assay (left) and quantification (right) of NSCLC cells upon GLI2 knockdown compared to backbone vector-transfected cells. **D** Spheroid formation assay (left) and quantification (right) of NSCLC cells upon exogenous GLI2 overexpression. Scale bar, 200 µm. **E** Sphere formation assay (left) and quantification (right) of NSCLC cells upon GLI2 repression. Scale bar, 200 µm. **F** Growth curve of NSCLC cells upon enforced MIR31HG upregulation or GLI2 repression in parallel. **G** Clonal formation assay (left) and quantification (right) of NSCLC cells upon MIR31HG-overexpressing and GLI2-knockdown double transfection. **H** Spheroid formation assay (left) and quantification (right) of NSCLC cells upon MIR31HG-overexpressing and GLI2-knockdown double transfection. Scale bar, 200 µm. Data are presented as mean ± SEM; significance abundance: *p < 0.05, **p < 0.01, ***p < 0.001.

To determine whether MIR31HG functions through GLI2, we first investigated the function of GLI2 regarding lung cancer stemness-related cellular proliferative advantage and drug resistance. Protein level detection by immunoblot, immunofluorescent staining, and flow cytometry demonstrated that upregulation of GLI2 led to overexpression of SOX2, ABCG2, CD133, and CD34, while GLI2 knockdown exerted inhibitory effect on above stemness-related targets (Fig. 5A and Fig. S6A–D). Chromatin immunoprecipitation (ChIP) results showed that GLI2 bound to the promoter region of SOX2 in NSCLC cells (Fig. S6, F). The clonal formation assay showed that exogenous GLI2 expression remarkably promoted cell growth while GLI2 repression prohibited cellular propagation (Fig. 5B, C). Spheroid culture and quantification also indicated increased growth of lung cancer stem cells upon GLI2 transfection and shRNA targeting GLI2 retarded spheroid formation of lung cancer cells (Fig. 5D, E). Drug sensitivity assay showed that GLI2 overexpression significantly (*p* < 0.01) enhanced drug resistance of lung cancer cells against targeted EGFR inhibitors and conventional chemotherapy drugs (Fig. S7A and Table S7). As the above data suggested that MIR31HG modulated the stemness-related cellular phenotype of lung cancer cells via GLI2, we performed rescue experiments by silencing GLI2 upon MIR31HG overexpression. Transfection efficacy of GLI2 knockdown in MIR31HG-overexpressing cells was validated by immunoblotting (Fig. S7B). Knockdown of GLI2 counteracted MIR31HG-induced cell proliferation, metastasis, and cancer stem cell-mediated multidrug resistance of lung cancer cells (Fig. 5F–H, and Fig. S7C). On the other side, exogenous GLI2 overexpression potently offset the inhibitory effect of MIR31HG knockdown on H50 cell proliferation and stem spheroid formation (Fig. S8A–C). These data suggested that MIR31HG promoted stemness features of lung cancer cells through positively modulation of GLI2 expression.

### Antisense oligonucleotide (ASO)-mediated MIR31HG-targeting therapy attenuates tumorigenesis and progression both in vitro and in vivo

**Fig. 6:**
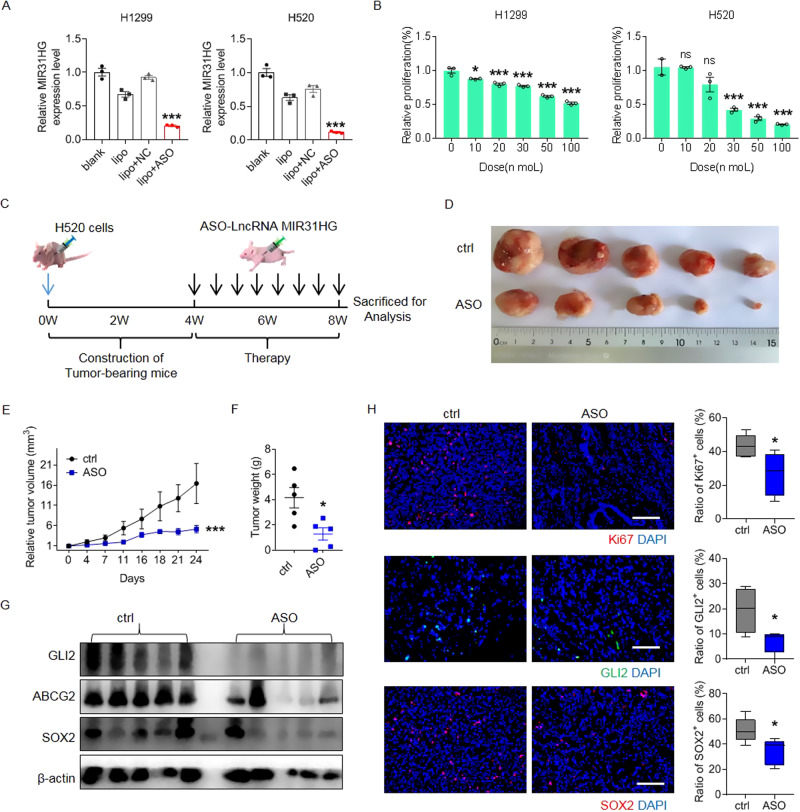
Antisense oligonucleotide drugs targeting MIR31HG attenuate lung cancer cell tumorigenesis both in vitro and in vivo. **A** Transcription levels of MIR31HG treated by ASO against MIR31HG and non-sense control detected by RT-PCR. blank: cells with no treatment; lipo: cells incubated with transfection reagent lipo3000; NC: negative-control antisense oligonucleotide; ASO: Antisense oligonucleotide specifically against MIR31HG. **B** Cell proliferative capability on treatment with the indicated dose of ASO-targeting MIR31HG or control measured by the CCK8 assay. **C** Schematic illustration of mice experiments. **D** Photographic documentation of tumor bulk treated upon ASO-targeting MIR31HG or control group. **E** Growth curve of tumor development of ASO-targeting MIR31HG or control group. **F** Weight of tumor bulk in ASO-targeting MIR31HG or control group after execution. **G** Protein levels of GLI2, ABCG2, and SOX2 in tumor bulk from different groups. **H** Immunofluorescent staining of Ki67, GLI2, SOX2 in tumor bulk from different groups and related quantification, respectively. Scale bar, 200 µm.

To explore the potential application of MIR31HG-targeting lung cancer therapy, commercially synthesized ASO performed functional analysis against MIR31HG in vitro and in vivo. Knockdown efficacy of ASO-targeting MIR31HG was validated by qPCR in H1299 (~80%) and H520 (~90%) cells (Fig. 6A). The CCK8 assay revealed that MIR31HG repression by ASO decreased cellular proliferation in a dose-dependent manner, particularly in H520 (Fig. 6B). Growth curve and clonal formation analyses further suggested that MIR31HG ASO treatment retarded lung cancer cell proliferation in vitro (Fig. S9A, B). Next, we tested the role of MIR31HG in tumor initiation and progression in vivo using a mouse model (Fig. 6C and S9C). MIR31HG ASO therapy significantly reduced tumorigenesis and development of H520 cells, whereas MIR31HG overexpression promoted tumor growth (Fig. 6D–F and S9D). qRT-PCR analysis displayed that MIR31HG ASO treatment downregulated transcription of MIR31HG, in tumor while MIR31HG overexpression had opposite effect (Fig. S9F). Immunoblot of tumor bulk protein and in situ immunohistochemistry indicated that MIR31HG ASO therapy downregulated expression of GLI2, ABCG2, and SOX2 (Fig. 6G, H, Fig. S10B, C). Ki67 staining of tumor bulk indicated that ASO therapy remarkably slowed cell proliferation while MIR31HG overexpression promoted Ki67 indicated cellular proliferation in vivo (Fig. 6H and Fig. S10A). Furthermore, ASO-mediated MIR31HG-targeting therapy significantly attenuated tumor metastasis in distant organs, including the lungs and liver, whereas MIR31HG overexpression remarkably promoted metastasis in vivo (Fig. S11A, B). To further verify that the metastatic neoplastic cells are indeed H520 cells implanted exogenous, Vimentin as a marker for H520 was stained in vitro and in vivo. Immunofluorescent staining indicated that compared to immortalized human epithelia HBEC cells, Vimentin was specifically expressed in H520 cells (Fig. S11C). Immunohistochemical staining of Vimentin in tumor, liver, and lung specimens displayed less metastasis upon ASO therapy and increased metastatic niche in overexpression groups (Fig. S11D, E).

## Discussion

The high morbidity and mortality associated with lung cancer calls for deeper investigation of the molecular mechanisms and identification of novel therapeutic approaches to overcome refractory multidrug resistance and insensitivity to immune therapy. Following previous work regarding the canonical activation of the Hedgehog cascade in cancer stemness-associated drug resistance and cellular invasion, we discovered in this study a non-canonical lncRNA MIR31HG dependent GLI2 stimulation and subsequent cancer stemness-related phenotypes. MIR31HG was overexpressed in lung cancer specimens and blood sera and correlated with a poor clinical outcome. Our mechanistic investigation revealed that MIR31HG promoted cancer stem cell-related malignant properties of lung cancer cells via H3 histone modification of GLI2 transcriptional activity and its downstream stemness-related molecules.

Extensive studies of MIR31HG in various cancer types have suggested conflicting functions and molecular mechanisms regarding tumor initiation and progression in a texture-based background. MIR31HG was found to be remarkably upregulated in tumor tissues of esophageal squamous cell carcinoma, pancreatic ductal adenocarcinoma, breast cancer, and basal subtype of bladder cancer, serving as a bona fide diagnostic indicator [10, 31–33]. In vitro and in vivo experiments have suggested oncogenic properties of MIR31HG overexpression in glioblastoma, oral cancer, osteosarcoma, and cervical carcinoma [34–37]. Mechanistic studies revealed that MIR31HG exerted its oncogenic role in versatile manners. MIR31HG promoted the invasiveness of cervical carcinoma cells by sponging miR-361-3p and positively modulate its target, epithelial membrane protein 1 (EMP1) [35]. In glioblastoma, MIR31HG could facilitate nucleus translocation and stimulate Wnt/β‑ catenin signaling cascade [37]. In addition, MIR31HG could recruit HIF-1α and its cofactors to targets genes and activate the HIF-1 transcriptional network in oral carcinoma [36]. In contrast, MIR31HG was found to be downregulated in gastric cancer hepatocellular carcinoma and esophageal squamous cell carcinoma, and this downregulation was associated with aggressive clinicopathological features and poor prognoses [38–41]. MIR31HG play a role of tumor suppressor in hepatocellular carcinoma by acting as a ceRNA to downregulate the oncogenic microRNA-575 [40]. The positive correlation between MIR31HG transcription, NSCLC cellular proliferation, and clonogenicity demonstrated in our study strongly suggests an oncogenic role of MIR31HG in NSCLC tumorigenicity and progression. Furthermore, our data displayed that MIR31HG modulated stem spheroid formation and stem-like cancer cells directed multidrug resistance by stimulate the Hedgehog pathway and downstream cascade, suggesting that MIR31HG and its downstream target may be candidate oncogenes for refractory lung cancer treatment. Overall, our findings suggest a complex role and an underlying molecular mechanism of MIR31HG in malignant transformation of lung cancer cells under various conditions.

A previous study found that MIR31HG regulated the differentiation of adipose-derived human stem cells by mediating enrichment of active H3K4me3 in the promoter of the adipogenic modulator [42]. However, we found that MIR31HG modulated the overall level of H3K4 mono-methylation, rather than di- or tri-methylation in lung cancer cells, suggesting a context-dependent histone modification. Although overall H3K4me3 detection could not exclude the possibility that MIR31HG might specifically influence the H3K4me3 modification in the promoter area of GLI2, predication of no complementary binding site between MIR31HG and GLI2 promoter area further diminished this possibility. Our data revealed that MIR31HG directly interacted with WDR5 to act as a scaffold, guiding the WDR5/MLL complex to GLI2 regulatory elements. Furthermore, H3K27 acetylation was closely modulated by MIR31HG at the same loci as H3K4me. The histone acetyltransferase P300/CBP complex control lysine 27 acetylation of histone H3 and positively regulate target gene expression by promoting enhancer activity [43, 44]. P300/CBP inhibition impairs H3K27 acetylation of enhancer regions across the genome of neoplastic cells and disrupts recruitment of essential transcriptional cofactors [45, 46]. In addition to the enhancer area, the P300/CBP complex mediates H3K27 acetylation in promoter regions of metabolic associated genes in hepatocellular carcinoma [47]. Corroborating this, we found that MIR31HG remarkably regulated both H3K4me and H3K27Ace modification in lung cancer cells. Co-localization of the modification loci of H3K4me/H3K27ace in regulatory elements of the GLI2-predicted enhancer area suggested a co-modulation mode for both factors. Furthermore, the observation that a single WDR5 or P300 inhibitor could independently suppress both H3K4me and H3K27ace suggests the existence of a complex that exerts both modifications. Protein interaction between WDR5/MLL3 and MLL3/P300 revealed a WDR5/MLL3/P300 complex that stabilized by MIR31HG in lung cancer cells. RIP data clearly demonstrated that the interaction between MLL3/P300 and MIR31HG was disrupted upon WDR5 inhibition, therefore corroborating the role of WDR5 as a scaffold protein between MLL3/P300 and MIR31HG. During physiological development of embryonic stem cells and malignant transformation of neoplastic cells, the WDR5/MLL/P300 complex coordinates to form a H3K4me+ H3Kace+ active landscape in the enhancer area across the genome [30, 48]. We found that WDR5 inhibitor impaired both H3K4me and H3K27ace modification, suggesting its indispensable role in H3K4me+ H3K27ace+ landscape formation. Whether MIR31HG guides the WDR5/MLL3/P300 complex throughout the whole genome for H3K4me + /H3Kace+ active landscape formation or only through a partial gene set or single gene remains unknown.

Cancer patients with Hedgehog pathway overactivation develop drug resistance to FDA-approved SMO inhibitors due to the compensatory SMO-independent, non-canonical activation of GLI1/2 [49]. GLI2 is reported to contribute to chemoresistance and stemness-related features in adenocarcinoma, pancreatic cancer, and colorectal cancer [50–52]. In addition to canonical SMO-dependent activation, GLI2 can be stimulated by aberrant expression of non-coding RNAs in malignant disorders [19]. Our previous study revealed that lncRNA SOX2OT exacerbated stemness features of lung cancer cells by guiding the METTL3/14 complex to facilitate m6A modification and stabilization of GLI1 RNA transcripts [53]. In the present study, we showed that GLI2 can be epigenetically upregulated at the transcriptional level by MIR31HG, exerting lung cancer cell stemness-related multidrug resistance, and cellular invasion.

**Fig. 7:**
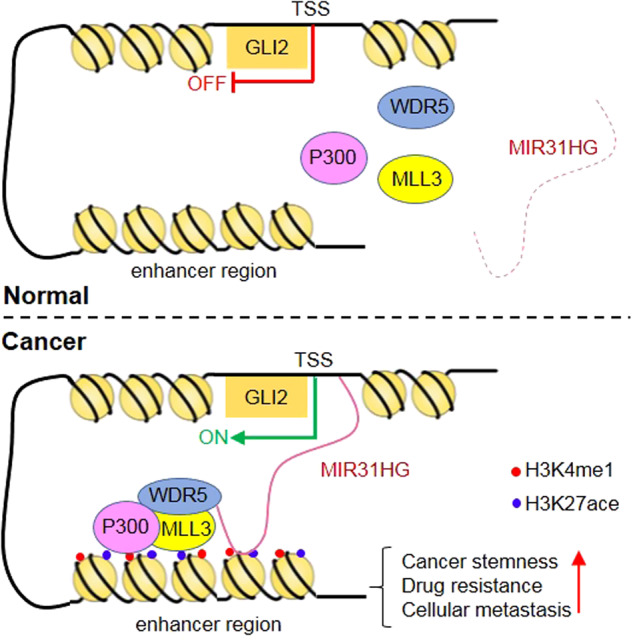
Mechanism of MIR31HG in NSCLC cells. In cancer cells, aberrantly overexpressed LncRNA MIR31HG acts as scaffold to guide WDR5/MLL/P300 complex to the enhancer region of GLI2 and initiated H3K4me/H3K27ace modification. Predicated binding sites between MIR31HG and GLI2 gene including one upstream TSS site and one in the enhancer area facilitate formation of a loop as indicated. The active H3K4me/H3K27ace landscape thus stimulated the transcription of GLI2 and its downstream target stemness-associated genes. Therefore, lead to stemness-related features.

In summary, the present work reported a novel mechanism of GLI2 activation that leads to NSCLC resistance in multiple therapeutic strategies, including chemotherapy, and targeting therapy. Our findings that lncRNA MIR31HG stimulates GLI2 by WDR5/MLL3/P300-mediated active chromatin landscape highlight lncRNA MIR31HG as a potential target for clinical lung cancer clinic treatment (Fig. 7). The present study is limited by the validation of combinational therapy by nucleic acid drugs targeting MIR31HG in immune-competent mice models due to the lack of the MIR31HG gene in mice; intervention of MIR31HG in immune system humanized lung cancer implanted mice models might reveal novel therapeutic candidates of NSCLC in the future.

## Methods

### Database analysis

Kaplan–Meier curves were generated by the Kaplan–Meier Plotter database based on data of lung cancer mRNA gene chips (Affymetrix ID: 1554097_a_at). Cox regression was performed to compute the hazard ratio (HR) and *p-value* using the univariate analyses [54]. MIR31HG expression in distinct NSCLC clinicopathological subtypes and stages was analyzed by Gene Expression Profiling Interactive Analysis (GEPIA) database using RNA sequencing expression data of NSCLC and normal samples from TCGA and the Genotype-Tissue Expression (GTEx) projects [55]. Bioinformatic analysis of H3K4me and H3K27Ace predicted regulatory elements on GLI2 using the University of California Santa Cruz (UCSC) genome browser based on data in seven cell lines in the ENCODE database (http://genome.ucsc.edu/) [56].

More detailed methods are available in the online-only Data Supplement.

### Supplementary information

Supplementary Table 1

Supplementary materials and methods

## Data Availability

All datasets generated and analyzed during this study are included in this published article and its Supplementary Information files. Additional data are available from the corresponding author on reasonable request.
