# Supplementary material for: LncRNA MIR31HG fosters stemness malignant features of non-small cell lung cancer via H3K4me1- and H3K27Ace-mediated GLI2 expression
Source: Oncogene. 2023 Nov 10;43(18):1328–40. doi: 10.1038/s41388-023-02883-4 (PMC11065682; doi:10.1038/s41388-023-02883-4)
Supplement: Supplementary file 1 — Supplementary Table 1 [file 41388_2023_2883_MOESM1_ESM.docx]

|  | **Sequence (5′-3′)** |
| --- | --- |
| P1 For | TCATCAATGGCAGAGGGA |
| P1 Rev | AGGCAGACCTGGGAACCT |
| P2 For | GAGAAGGCGGAATGAATA |
| P2 Rev | GAAGGAAATGTGCCTCTTA |
| P3 For | TGCAGCCACCGTTAGGACA |
| P3 Rev | ACATGGGAAGGGTTTATG |
| P4 For | ACTTAACCAGCCCCTTAT |
| P4 Rev | AAGTTCTCACTCTGACCCA |
| P5 For | AGCCAGTCTTAGAGCGTA |
| P5 Rev | GGGTGACAGGATGCCACA |
| P6 For | GGCTCCTCATTCAGGCACT |
| P6 Rev | ACCCAAGCACGCAACAGA |
| P7 For | GCTGTGGGGTGAGGGAAGT |
| P7 Rev | TCCAACACCGCTGAAGCA |
| P8 For | GGAGCTTGCTGGGTTGTGCC |
| P8 Rev | AAGGAGGGAGGGAAGGAGGG |
| P9 For | ATCCACTACCCTTCACTTAC |
| P9 Rev | CCAGACTCTGGGTTGAGAT |
| P10 For | GCTTTGGGTGCATTTGAG |
| P10 Rev | GGTTTAGATAGTGGTGGTGGT |
| N1 For | CACCTGGCCCTATCTTTA |
| N1 Rev | ACGGCAGTGGTACGAAAA |
| N2 For | CTGGGATTATAGGCGTCC |
| N2 Rev | CAGCACTTTCAGAGGTCG |
| N3 For | TAGAAGTTTACACTCCCACC |
| N3 Rev | AACTCCGCTGAAACATAG |

**Table S1.** Primers used for Cut&Tag-qPCR to amplify the enhancer region of GLI2.

**Table S2.** Primers used for Cut&Tag-qPCR to amplify the promoter region of SOX2.

| **Primer for SOX2 promoter** | **Sequence (5′-3′)** |
| --- | --- |
| P1 For | CCTAATCTCCAGGTCCGTGTTT |
| P1 Rev | CGGCGCTCTTCAAAATACAGC |
| P2 For | GTTTGACAGTAACAGGCTAGGGA |
| P2 Rev | CTTCCCATAATCACTCCCCCG |
| P3 For | AGCCACAAAGATCCCAACAAGA |
| P3 Rev | GTTGTCGCTACACGGAGTCA |
| P4 For | CGGCCACCACAATGGAAATCTA |
| P4 Rev | CTCCCTCCCACGCAGAGTT |
| P5 For | GAAACCCTTCTTACGGGGAGG |
| P5 Rev | CAACCGTAGCAAAGGGGATG |
| P6 For | CTTACCAAGGCCTGCTGGTT |
| P6 Rev | CCTTGCTTCCACGTAACTTGC |
| P7 For | GCACCTGTAAGGTAAGAGAGGA |
| P7 Rev | TGTTCTCCCGCTCATCCACA |
| P8For | TTTATTCCCTGACAGCCCCC |
| P8Rev | CTCTGCCTTGACAACTCCTGA |

**Table S3.** Primers used for RT-QPCR to amplify the mRNA of target genes.

| **Primer name** | **Sequence (5′-3′)** |
| --- | --- |
| MIR31 For | CAGGCAAGATGCTGGCATAGC |
| MIR31 Rev | TGGTGTCGTGGAGTCG |
| CD34 For | GTCTTCCACTCGGTGCGTCT |
| CD34 Rev | TGGGGTAGCAGTACCGTTGT |
| CD133 For | TTGATCCGGGTTCTTACCTG |
| CD133 Rev | GCTTTGCAATCTCCCTGTTG |
| CD44 For | GCAACTCCTAGTAGTACAACGGAAGA |
| CD44 Rev | CGATATCCCTCATGCCATCTGA |
| GLI2 For | CTGTGGGTTAGGGATGGACTGA |
| GLI2 Rev | CACGCAACTTCCTCTTCCTACTGA |
| ABCG2 For | GTAATCCCCAGGCCTCTATAG |
| ABCG2 Rev | ACTTGGTAACATCCTCATGGG |
| SOX2 For | GGGAAATGGGAGGGGTGCAAAAGA |
| SOX2 Rev | TTGCGTGAGTGTGGATGGGATTGG​ |
| GAPDH For | CTCCTCCTGTTCGACAGTCAGC |
| GAPDH Rev | CCCAATACGACCAAATCCGTT |
| MIR31HG For | CAAGCAGGTCTCCAGGTGTT |
| MIR31HG Rev | CCAGGCTATGTCTTTCCTCTAT |

**Table S4.** Sequence of MIR31HG KD #1, KD #3 and MIR31HG ASO.

| **Name** | **Sequence** |
| --- | --- |
| h-MIR31HG KD #1 | TGGTTCTGAGGCAGGTTATATCTCGAGATATAACCTGCCTCAGAACCA |
| h-MIR31HG KD #3 | TGATGATTCACAAGGTATTTACTCGAGTAAATACCTTGTGAATCATCA |
| ASO-h-MIR31HG | AAGGAGGTCATCACTCTACA |

**Table S5.** IC50 and RI of the four indicated drugs in lung cancer cells upon MIR31HG overexpression

| **Cells** | **Drug** | **IC50(pc3.1)** | **IC50(MRI31HG)** | **RI** | **P value** |
| --- | --- | --- | --- | --- | --- |
| H1299 | DDP | 5.078 | 6.969 | 1.372 | <0.0001 |
|  | GEM | 14.310 | 28.170 | 1.969 | <0.0001 |
|  | Gefitinib | 31.370 | 52.280 | 1.667 | <0.0001 |
|  | Osimertinib | 6.624 | 13.250 | 2.000 | <0.0001 |
| H520 | DDP | 3.576 | 5.594 | 1.564 | <0.0001 |
|  | GEM | 10.540 | 35.200 | 3.340 | <0.0001 |
|  | Gefitinib | 14.390 | 29.240 | 2.032 | <0.0001 |
|  | Osimertinib | 5.746 | 7.866 | 1.369 | <0.0001 |

**Table S6.** IC50 and RI of the four indicated drugs in lung cancer cells upon MIR31HG repression.

| **Cells** | **Drug** | **IC50 (shv)** | **IC50 (#1)** | **RI** | **P value** |
| --- | --- | --- | --- | --- | --- |
| H1299 | DDP | 13.000 | 6.482 | 0.499 | <0.0001 |
|  | GEM | 10.700 | 5.643 | 0.527 | <0.0001 |
|  | Gefitinib | 30.480 | 20.840 | 0.684 | <0.0001 |
|  | Osimertinib | 13.080 | 8.952 | 0.684 | <0.0001 |
| H520 | DDP | 11.170 | 5.982 | 0.536 | <0.0001 |
|  | GEM | 30.770 | 0.962 | 0.0313 | <0.0001 |
|  | Gefitinib | 31.26 | 16.15 | 0.517 | <0.0001 |
|  | Osimertinib | 8.952 | 3.691 | 0.412 | <0.0001 |

**Table S7.** IC50 and RI of indicated four drugs in lung cancer cells upon GLI2 overexpression.

| **Cells** | **Drug** | **Ctrl**  **IC50** | **GLI2**  **IC50** | **RI** | **P value** |
| --- | --- | --- | --- | --- | --- |
| H1299 | DDP | 3.059 | 4.174 | 1.364 | P<0.001 |
|  | GEM | 14.780 | 17.500 | 1.1840 | P<0.0001 |
|  | Gefitinib | 10.82 | 20.91 | 1.933 | P<0.0001 |
|  | Osimertinib | 7.713 | 10.310 | 1.337 | P<0.0001 |
| H520 | DDP | 5.541 | 7.000 | 1.263 | P<0.001 |
|  | GEM | 4.923 | 12.760 | 2.592 | P<0.0001 |
|  | Gefitinib | 16.290 | 21.180 | 1.300 | P<0.0001 |
|  | Osimertinib | 5.789 | 8.786 | 1.518 | P<0.0001 |
