# Supplementary material for: LncRNA MIR31HG fosters stemness malignant features of non-small cell lung cancer via H3K4me1- and H3K27Ace-mediated GLI2 expression
Source: Oncogene. 2023 Nov 10;43(18):1328–40. doi: 10.1038/s41388-023-02883-4 (PMC11065682; doi:10.1038/s41388-023-02883-4)
Supplement: Supplementary file 2 — Supplementary materials and methods [file 41388_2023_2883_MOESM2_ESM.pdf]

Supplementary Materials for

LncRNA MIR31HG fosters stemness malignant features of non-small cell lung cancer via H3K4me1- and H3K27Ace-mediated GLI2 expression

Weiwei Chen[1†], Fei Wang[1†], Xinyuan Yu[2†], Jingjing Qi[3], Hongliang Dong[1], Bingjie Cui[1], Qian Zhang[4], Yan Wu[1,2], Jiajia An[5], Na Ni[1], Cuilan Liu[1], Yuchen Han[5], Shuo Zhang[1,6], Clemens A. Schmitt[7,8,9,10,11], Jiong Deng[1]*, Yong Yu[3]*, Jing Du[1,2,6]*

Methods

Cell culture

The NSCLC cell lines A549 and H1299 were obtained from the American Type Culture Collection (ATCC). HBEC, H520, and SW900 cells were maintained in our lab. The NSCLC cell lines were cultured in RPMI1640 medium (01-100-1ACS, Biological Industries, Beit HaEmek, Israel) supplemented with 10% fetal bovine serum (FBS; C04001-500, VivaCell Biosciences, Shanghai, China) and 100 μg/mL penicillin and streptomycin (B540732-0010, Sangon Biotech, Shanghai, China). All cells were incubated at 37 °C with 5% CO_2 and passaged every alternate day at a 1:3 ratio.

For cell treatments, 0, 1, 2, 4, 8, and 16 μM of cisplatin (DDP; MedChemExpress, Monmouth Junction, NJ, USA; HY-17394) and gemcitabine (GEM; HY-17026, MedChemExpress) were added. Gefitinib (HY-50895, MedChemExpress) and osimertinib (B011037, Energy Chemical, Anhui Zesheng Technology Co., Anhui, China, LTD) solutions were added at 0, 3.125, 6.25, 12.5, and 50 μM concentrations (Tables S5-S7 and Fig. S2). The working concentrations of WDR5 inhibitor OICR-9429 (HY-16993, MedChemExpress) were 0, 10, 20, and 40 μM. The working concentrations of MLL1 inhibitor MM-102 (HY-12220, MedChemExpress) were 0, 10, 20, and 40 μM. The working concentrations of P300 inhibitor C646 (S7152, Selleck Chemicals, Houston, TX, USA) were 0, 12.5, 25, and 50 μM. After 48 h, the cells were collected for further analysis.

Plasmids and transfection

The pcDNA 3.1-MIR31HG (MIR31HG) plasmid was constructed by our laboratory. The MIR31HG knockdown plasmids pLV[shRNA]-EGFP:T2A:Puro-U6 (#1 and #3) were constructed by Cyagen Biotechnology (Shanghai, China), the GLI2 overexpression plasmid GV492 was constructed by Genechem Biotechnology (Shanghai, China), and the GLI2 knockdown plasmids pGPU6 (#1 and #2) were constructed by GenePharma Biotechnology (Shanghai, China). All plasmids were extracted using the AxyPrep Plasmid Miniprep Kit following manufacturer instructions (AP-MN-P-250, Corning, Corning, NY, USA).

H1299, and H520 cells were seeded in six-well plates at a density of 3×10^5 cells per well and transiently transfected using Lipofectamine 3000 reagent (L3000015, Thermo Fisher Scientific, Waltham, MA, USA) according to manufacturer instructions. Antibiotic selection was performed using 400 μg/mL G418 (HY-17561, MedChemExpress, Monmouth Junction, NJ, USA) for 14 d or 1 μg/mL puromycin (HY-1569, MedChemExpress5) for 5 d for stable transfection.

Proliferative assay

H1299 and H520 cells were seeded into a 96-well plate at a density of 3000 cells per well at 24 h before drug treatment. After 48 h of drug incubation, the proliferative capability of cells was determined using the MCE Cell Counting Kit-8 (HY-K0301, CCK-8; MedChemExpress) according to manufacturer instructions (Table S5-S7). The absorption and reference wavelengths were measured using a BioTek Synergy H1 hybrid microplate reader at 450 and 630 nm, respectively. The IC50 of the drugs were calculated by the Graphpad Prism Software package 8.0 (San Diego, CA, USA) based on the absorbance values of five geometric multiple doses in addition to vehicle control without any drug treatment other than CCK8 (MedChemExpress).

Colony formation assay

Cells were seeded into a six-well plate at a density of 700 cells per well and cultured for 14 d in RPMI1640 medium. Thereafter, cells were fixed with 4% paraformaldehyde

(A500684, Sangon Biotech) and stained with crystal violet (A100528, Sangon Biotech) for 15 min before optical imaging using an Olympus IX53 inverted fluorescence microscope paired with an Olympus DP73 color camera (Olympus, Tokyo, Japan). Molecular imager (ChemiDocXRS+, Bio-Rad, Hercules, CA, USA) was used to screen and analyze the densitometry of each visual field.

Wound healing assay

Cells were seeded into 12-well plate at a density of 1×10^5 cells per well for 24 h. When cells reached 90% confluence, a gap was created using a 200 μL tip of a pipette and washed with phosphate-buffered saline (PBS). Cells were incubated with serum-free medium for 24 h before photomicrographs were taken with the IX53 inverted microscope and DP73 color camera (Olympus). Relative distance of the remaining wound area was calculated as previously described [1]. Experiments were performed in triplicate.

Transwell assay

A transwell migration assay was performed as previously described [2]. Briefly, 1×10^5 cells were suspended in 200 μL of RPMI 1640 medium (Biological Industries, Beit-Haemek, Israel) without FBS and seeded on the top chamber of transwell inserts (3422, Corning). The lower chambers contained RPMI1640 medium with FBS. After incubation for 48 h, the chamber was rinsed three times with PBS, then dyed with crystal violet for 30 s and rinsed again with PBS five times to obtain a transparent background. Plates were imaged using the IX53 inverted fluorescence microscope and the DP73 color camera (Olympus).

Sphere formation assay

Cells were cultured in serum-free RPMI1640 medium supplemented with 20 ng/mL fibroblast growth factor (FGF; AF-100-17A, Peprotech, Cranbury, NJ, USA), 20 ng/mL epidermal growth factor (EGF; Peprotech; 100-47), and 2% B27 (17504-044, Thermo Fisher Scientific). The medium was 50% replaced every 3 d. Spheroids were

photographed and counted using the IX53 inverted fluorescence microscope and DP73 color camera (Olympus) 14 d later.

Immunofluorescence staining

Cells were seeded in 24-well plates at 5×10^4 per well. After 24 h, cells were fixed as previously described [2]. Cells were incubated with primary antibodies overnight at 4°C. As a negative control, slides incubated with PBS were stained in parallel. Then cells were incubated with the secondary antibody Alexa Fluor 594 AffiniPure Goat Anti-Rabbit IgG (H+L; 33112ES60, Yeasen Biotechnology Co., Ltd., Shanghai, China) and diluted with PBS for 2 h at 37 °C. Cells were counterstained with 4',6-diamidino-2-phenylindole (DAPI) and visualized with an advanced fluorescence microscope (Olympus BX63).

Anti-CD34 (A23107) and anti-CD133 (A0219) antibodies were purchased from ABclonal, Wuhan, China. Anti-vimentin (YT4879) antibody was purchased from ImmunoWay Biotechnology Company, Plano, TX, USA.

Co-IP

H1299 and H520 cells were collected and lysed for 30 min on ice. A part of the centrifuged lysate was used as an input sample. The other part of the lysate was mixed with 5 μg antibody and 30 μL rProtein A/G MagPoly Beads (SM015005, Changzhou Smart-Lifesciences Biotechnology Co., Ltd.) and vortexed for 3 h at 4°C. The mixture was washed five times with TBST. Subsequently, the mixture was inoculated with 25 μL protein loading buffer and boiled for 10 min at 95 °C. Samples were centrifuged and stored at -80 °C for further analysis. Antibodies against WDR5 (#13105, Cell Signaling Technology, Danvers, Massachusetts, USA), mono-methyl-histone H3 (#5326, Cell Signaling Technology), MLL3 (28437-1-AP, Proteintech, Wuhan, China) and P300 (A13016, Abclonal, Woburn, MA, USA) were used for protein immunoprecipitation.

RIP assay

H1299 and H520 cells were either treated with 20 μM WDR5 inhibitor OICR-9429

(HY-16993/CS-5776, MedChemExpress) or left untreated for 48 h; the procedure was identical to that subsequent for the control and experimental groups. All cell precipitates were chilled in polysome lysis buffer for 5-10 min on ice. All lysates were centrifuged at 13,000 g for 10 min; some supernatant was preserved as an input sample, and the rest was treated with antibody-rProtein A/G MagPoly Beads (SM015005, Changzhou Smart-Lifesciences Biotechnology Co., Ltd., Changzhou, China) mixture, which was preincubated at 25℃ for 30 min and vortexed at 4 °C overnight. Samples were washed six times with NT-2 buffer and further treated with proteinase K buffer (MedChemExpress; HY-108717). After heating in a water bath at 55 °C for 30 min, the RNA was extracted at 5,000 g for 15 s using a mini centrifuge (D1008E, DLAB Scientific, Beijing, China) and by collecting the supernatant of the NT-2 buffer. The antibodies used in the above experiments are those against WDR5 (#13105, Cell Signaling Technology), MLL3 (28437-1-AP, Proteintech) and P300 (A13016, Abclonal, Wuhan, China) that were used for MIR31HG immunoprecipitation.

CUT&Tag

This experiment was conducted according to the instructions of the NovoNGS® CUT&Tag 2.0 High-Sensitivity Kit (N259-YH01-01B, Novoprotein Scientific, Suzhou, China). Primary antibodies specific to GLI2 (YN3016, ImmunoWay), IgG (N269-01A, Novoprotein Scientific), mono-methyl-histone H3 (#5326, Cell Signaling Technology) and acetyl-histone H3 (#8173, Cell Signaling Technology) were used for chromatin immunoprecipitation, respectively. Primer sequences for the enhancer and promoter of GLI2 and SOX2 are listed in Table S1 and Table S2, respectively.

ChIP

This experiment was conducted according to the instructions of the ChIP-IT High Sensitivity Kit (53040, Active Motif, Carlsbad, CA, USA). A primary antibody specific to WDR5 (#13105, Cell Signaling Technology, Danvers, Massachusetts, USA), MLL3 (28437-1-AP, Proteintech, Wuhan, China) P300 (A13016, Abclonal, Woburn, MA, USA) and IgG (N269-01A, Novoprotein Scientific) were used for chromatin

immunoprecipitation. And primer sequences for the enhancer of GLI2 are listed in Table S1.

Immunoblot analysis

Cells were collected and lysed with RIPA lysis buffer (P0013B, Beyotime, Jiangsu, China) containing phenylmethanesulfonyl fluoride (Beyotime; ST506) on ice. After centrifuging at 12,700 rpm for 15 min, supernatant was harvested, and the protein concentration was determined using a BCA Kit (P0012, Beyotime) following manufacturer instructions. Proteins were separated by sodium dodecyl-sulfate polyacrylamide gel electrophoresis (SDS-PAGE) and electroblotted on polyvinylidene difluoride (PVDF) membrane (ISEQ00010, Millipore, Burlington, MA, USA). Membranes were incubated with primary antibodies at 4 °C for 12 h, then incubated with secondary antibodies for 2 h at 37 °C. Enhanced chemiluminescence signals were detected using Molecular imager (ChemiDocXRS+, Bio-Rad). Antibody against tubulin (#5568, Cell Signaling Technology) was used in parallel as an internal control.

Primary antibodies specific to GLI2 (YN3016) and SOX2 (YM0953) were purchased from ImmunoWay. Antibody against ABCG2 (BM4922) was purchased from Boster Bio (Pleasanton, CA, USA). Antibody against PD-L1 (66248-1-Ig) was obtained from Proteintech. Antibodies against WDR5 (#13105), tri-methyl-histone H3 (#9751), di-methyl-histone H3 (#9725), mono-methyl-histone H3 (#5326), acetyl-histone H3 (#8173) and tubulin (#5568) were purchased from Cell Signaling Technology. Secondary antibodies HRP-linked goat anti-rabbit IgG (H+L; #7074) and goat anti-mouse IgG (H+L; #14709) were also obtained from Cell Signaling Technology.

RNA isolation and real-time qPCR (qRT-PCR)

Total RNA of cultured cells was isolated using a Total RNA Isolation Kit (Shanghai Promega Biological, Beijing, China) following manufacturer's instructions. Blood samples from healthy and NSCLC patients were obtained from Binzhou Medical University Hospital in accordance with a protocol approved by the Review Board

(Approval No.: KYLL-2021-05). lncRNA was extracted from sera using a BIOG cfRNAEasy Kit (51028, Changzhou Bio-generating Biotechnology Corp, (Guangzhou, China). A RevertAid First Strand cDNA Synthesis Kit (K1622, Thermo Fisher Scientific) was used for cDNA synthesis. The concentrations of cDNA in different groups were measured and adjusted for qPCR. Real-time qPCR was performed on a CFX96™ Real-Time PCR System (1855195, Bio-Rad) with a 20 μL reaction pool, including 10 μL FastStart Universal SYBR Green Master (Novoprotein), 1 μL forward primer (Sangon Biotech), 2 μL cDNA, 1 μL reverse primer, and 6 μL deionized DEPC water. PCR conditions were as follows: 95 °C, 10 s; 95 °C, 5 s, 54 °C, 34 s, 40 cycles. Gene expression was normalized to that of GAPDH in the quantification analysis. All primer sequences used in this study are shown in Table S3.

In vitro RNA pull-down assay

This experiment was conducted according to the instructions of the BersinBio RNA pulldown Kit (Bes5102, BersinBio, Guangzhou, China). Biotin-labeled RNA was synthesized in vitro using Ribo^{TM}RNAmax-T7 biotin-labeled transcription kit (C11002-1, Ribbio, Guangzhou, China). The MIR31HG Sense primer sequences: For, 5′-TAATACGACTCACTATAGGGAGGTTCCACGTCCGGCGCCT-3′ and Rev, 5′-GACAAAGGTCACAAAGGATT-3′, Antisense primer sequences: For, 5′-TAATACGACTCACTATAGGGGACAAAGGTCACAAAGGATT-3′ and Rev, 5′-AGGTTCCACGTCCGGCGCCT-3′.

Flow cytometry analysis

Stably transfected MIR31HG overexpression and knockdown cells, GLI2 overexpression and knockdown cells were harvested, filtered, and centrifuged. anti-CD34 (A23107) and anti-CD133 (A0219) (ABclonal, Wuhan, China) antibodies were used for surface staining overnight at 4 °C. After washing with phosphate-bufered saline (PBS) for three times, adding fluorescein secondary antibody and incubating at room temperature for 1 h. After washing with PBS buffer for three times and finally resuspended in PBS buffer for surface marker detection On the BD C6 Flow C6 flow

meter and analyzed in FlowJo software.

Tissue microarray and probe hybridization

cDNA from 15 pairs of human NSCLC tissues and adjacent lung specimens were obtained from Shanghai Outdo Biotech (cDNA-HLugA030PT01) for further real-time qPCR analysis. A tissue microarray (HLug-Squ150Sur-01) was purchased from Shanghai Outdo Biotech Co., Ltd. (Shanghai, China), who also performed probe hybridization to detect the transcription levels of lncRNA-MIR31HG in 75 paired NSCLC tissues and adjacent specimens. Probes detecting MIR31HG were synthesized according to manufacturer's instructions for the Enhanced Sensitive ISH Detection Kit Ⅰ (MK1030, Boster Bio, Wuhan, China).

Tumor-bearing mouse model construction and treatments

Four-week-old BALB/c nude mice (female, 15-18 g) were purchased from Ji'nan Pengyue Laboratory Animal Breeding Co., Ltd. (Jinan, China) and kept in a specific pathogen-free laboratory animal room. The use of animals was approved by the Animal Experiments Ethical Committee of Binzhou Medical University Hospital and performed according to the Guide for Care and Use of Laboratory Animals. A tumor-bearing mouse model was developed via subcutaneous injection of 5×10^6 H520 cells per mouse. When tumors reached approximately 5 mm in diameter, mice were randomly divided into an H520 control group, an H520 MIR31HG overexpression group, an ASO control group, and an ASO-MIR31HG group for subsequent treatment (n = 5 in each group). To evaluate the therapeutic potential of targeting MIR31HG with ASO, 5 nmol of ASO-hMIR31HG-001 (Ribobio, Guangzhou, China; lnc6211215095815) were dissolved in 50 μL RNase free ddH_2O and administered via intra-tumoral injection every 2 d for 21 d. ASO control group mice were injected with saline. The tumor size and body weight of mice were measured every alternate day. Tumor volume was calculated as previously described [1]. All mice were euthanized after 4 weeks of treatment and tumors were excised for further analysis.

Histological analysis

Hematoxylin and eosin and immunohistochemistry staining of paraffin sections were performed as previously reported [1]. Images were observed via bright field microscopy (BX63, Olympus) and quantified using Image J software. For immunofluorescence staining, tissues were immersed in 30% sucrose solution overnight and embedded in Tissue Tek OCT compound (4583, Sakura Finetek, Tokyo, Japan). Subsequently, frozen sections (5 μm) were prepared using a cryostat (Leica CM1950, Leica, Wetzlar, Germany). Frozen sections were incubated with 5% normal goat serum (Zhongshan Golden Bridge Biotechnology, Beijing, China) for 40 min at 37°C. Primary antibodies against SOX2 (A0561, Abclonal) and Ki67 (A16919, Abclonal) were diluted 1:100 in PBS, applied to cover sections, and incubated at 4°C for 12 h. Sections incubated with only PBS were used as negative controls. Sections were then incubated with secondary antibody Alexa Fluor 594 AffiniPure Goat Anti-Rabbit IgG (H+L; 33112ES60, Yeasen Biotechnology Co., Ltd.) in PBS for 2 h at 37°C. DAPI was used to co-stain the cell nuclei. Images were acquired under an advanced fluorescence microscope (BX63, Olympus BX63) and quantified using Image J software.

Quantification and statistical analysis

Data are presented as mean ± standard error of mean (SEM). Graphpad Prism Software 8.0 (San Diego, CA, USA) was used for statistical analysis. Comparisons of two groups were analyzed with two-tailed Student’s *t*-test. Multiple comparisons were performed using one-way analysis of variance (ANOVA) or two-way ANOVA. Differences were considered significant at $p < 0.05$.

1. Du J, Chen W, Yang L, Dai J, Guo J, Wu Y, et al. Disruption of SHH signaling cascade by SBE attenuates lung cancer progression and sensitizes DDP treatment. Sci Rep. 2017;7(1):1899.
2. Chen W, An J, Guo J, Wu Y, Yang L, Dai J, et al. Sodium selenite attenuates lung adenocarcinoma progression by repressing SOX2-mediated stemness. Cancer Chemother Pharmacol. 2018;81(5):885-895.

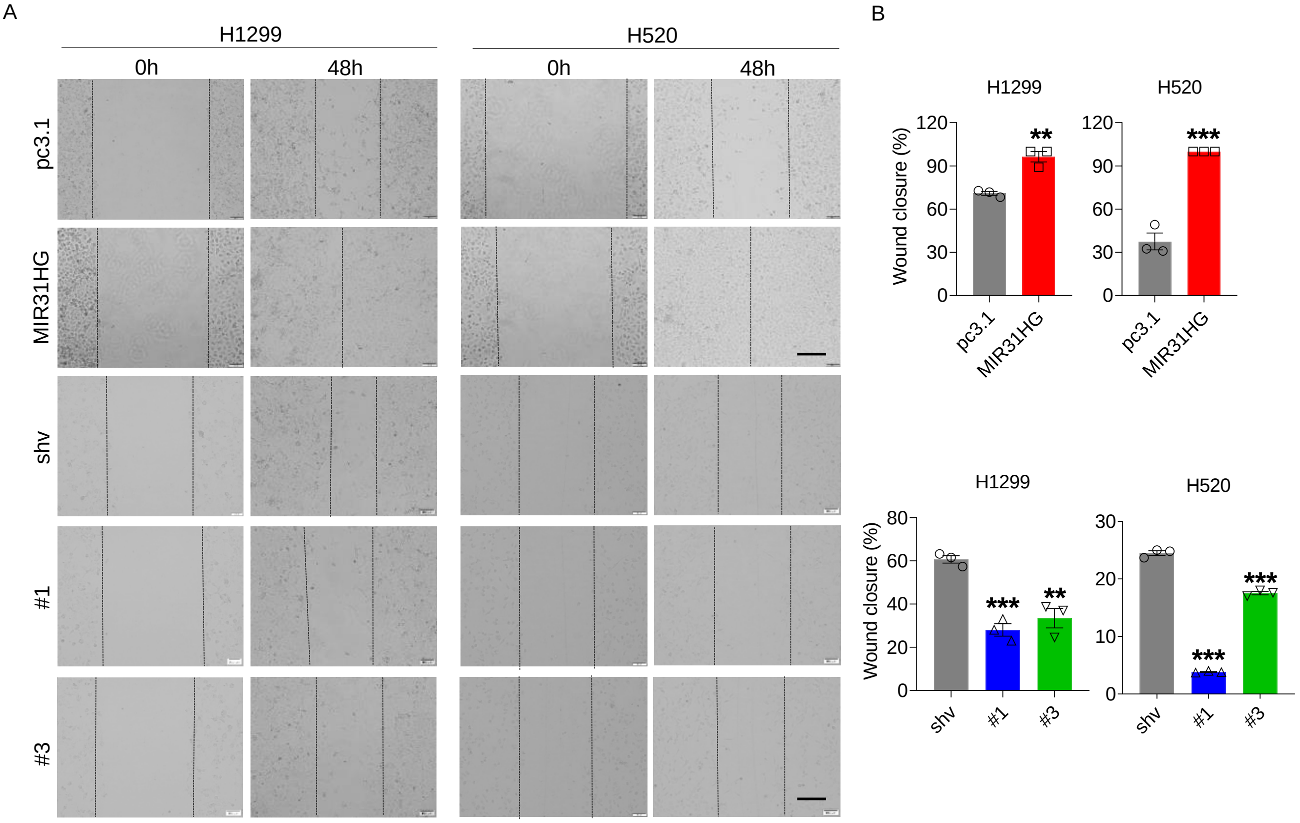

Fig. S1 MIR31HG promotes migration capability in NSCLC cells. A, B Wound healing assay (left) and histogram quantification (right) of H1299 and H520 cells upon forced MIR31HG overexpression or repression, compared to pc3.1 and shv empty vector separately. Scale bar, 200 µm. Data are presented as mean ± SEM; significance abundance: * $p < 0.05$, ** $p < 0.01$, *** $p < 0.001$.

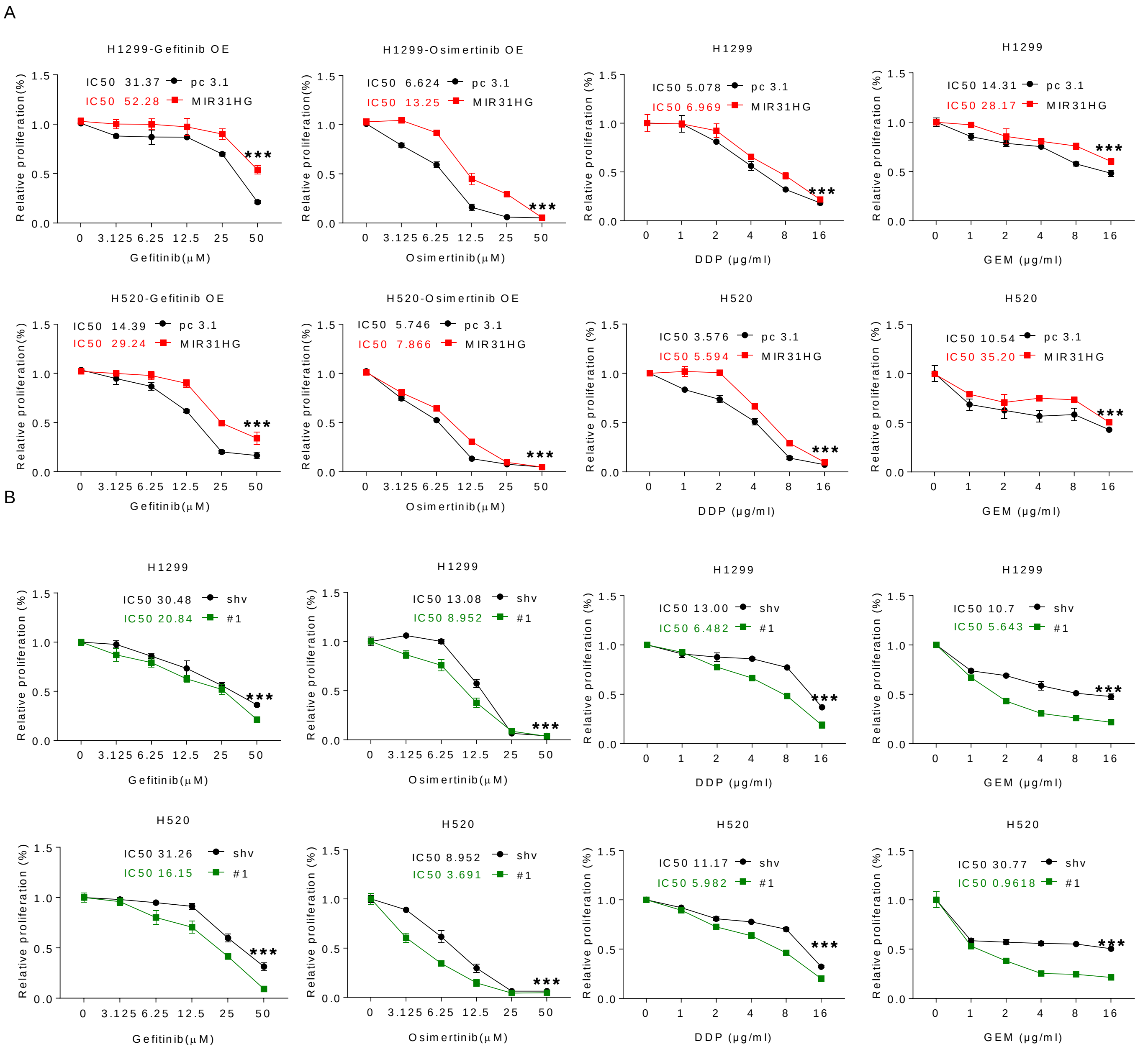

Fig. S2 MIR31HG promotes multidrug resistance in NSCLC cells. A Growth curve of NSCLC cells treated with gefitinib, osimertinib, DDP, or GEM uder MIR31HG overexpression in H1299 (upper panel) and H520 (lower panel) cells, as detected by the CCK8 assay. pc 3.1: pcDNA3.1 backbone; MIR31HG: pcDNA3.1-MIR31HG. DDP: cisplatin; GEM: gemcitabine. **B** Growth curve of NSCLC cells treated with gefitinib, osimertinib, DDP, or GEM under MIR31HG genetic inhibition in H1299 (upper panel) and H520 (lower panel) cells, as detected by the CCK8 assay. shv: pLV backbone; #1: pLV-MIR31HG shRNA. Data are presented as mean ± SEM; significance abundance: * $p < 0.05$, ** $p < 0.01$, *** $p < 0.001$.

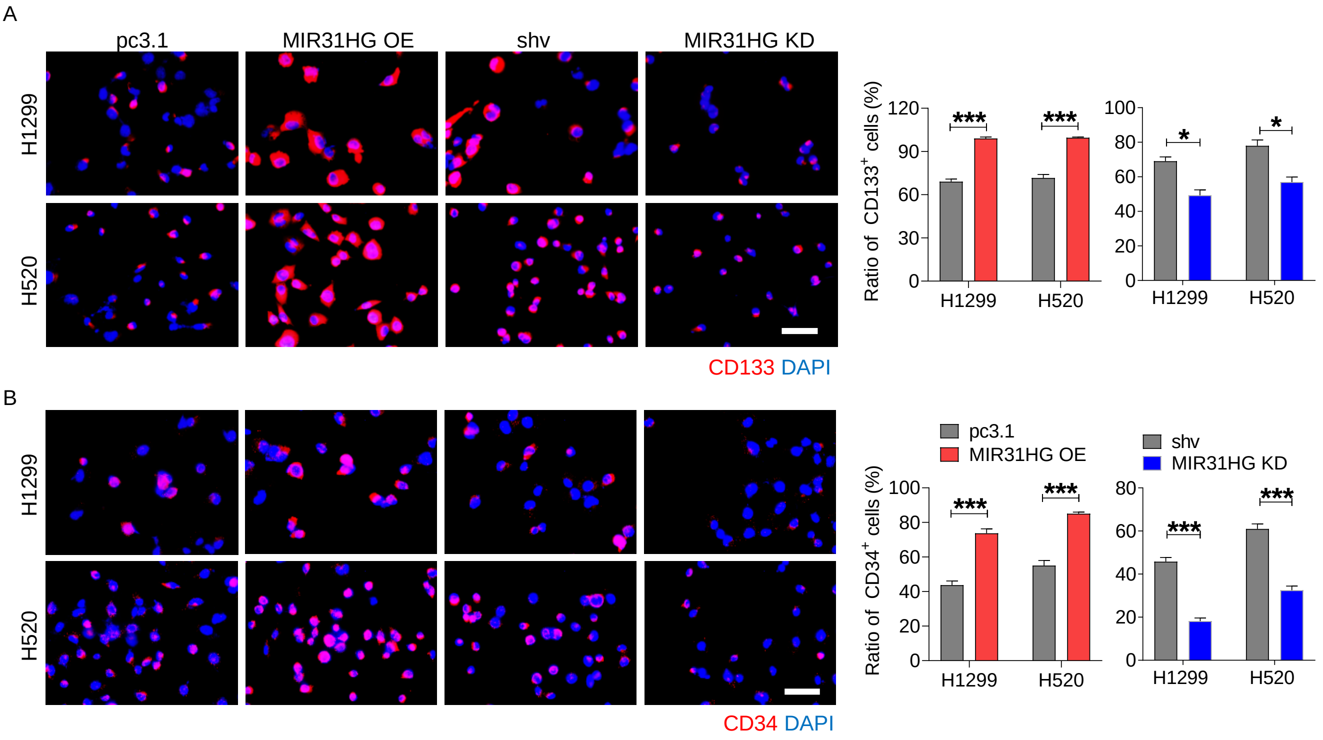

Fig. S3 MIR31HG promotes expression of stem cell markers in NSCLC cells. A, B Immunofluorescent staining (left) and histogram quantification (right) of CD133 and CD34 in H1299 and H520 cells upon forced MIR31HG overexpression or repression, compared to pc3.1 and shv empty vector separately. Scale bar, 100 μm. Data are presented as mean ± SEM; significance abundance: * $p < 0.05$, ** $p < 0.01$, *** $p < 0.001$.

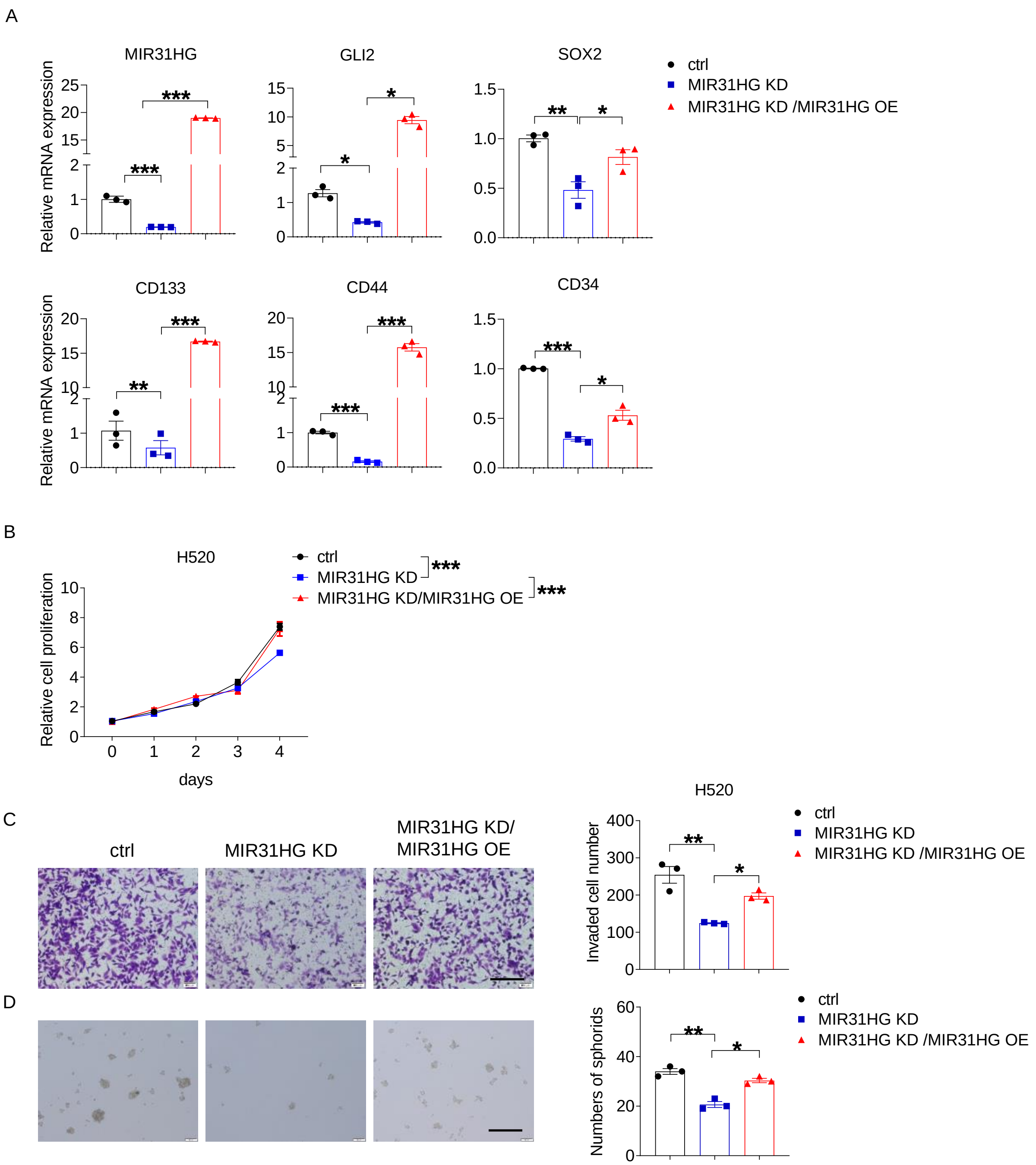

Fig. S4 MIR31HG overexpression reverses the effect caused by knockdown of MIR31HG in lung cancer cells in NSCLC H520 cells. A Transcriptional levels of MIR31HG, GLI2, SOX2, CD133, CD44, CD34 upon shv, MIR31HG knockdown, MIR31HG overexpression and MIR31HG knockdown double transfection respectively, as detected by qRT-PCR. **B** Growth curve of shv, MIR31HG knockdown, MIR31HG overexpression and MIR31HG knockdown double transfection by the CCK8 assay in H520 cells. **C** Invasive capability (left) and quantification (right) of shv, MIR31HG knockdown, MIR31HG overexpression and MIR31HG knockdown double transfection in H520 cells, as detected by transwell staining and histogram quantification, respectively. Scale bar, 200 µm. **D** Spheroid formation assay (left) and quantification (right) of H520 cells upon MIR31HG overexpression and MIR31HG knockdown double transfection. Scale bar, 200 µm. shv: pLV backbone; MIR31HG KD: pLV-MIR31HG shRNA #1; MIR31HG OE: pcDNA3.1 based MIR31HG overexpressing plasmid . Data are presented as mean ± SEM; significance abundance: * $p < 0.05$, ** $p < 0.01$, *** $p < 0.001$.

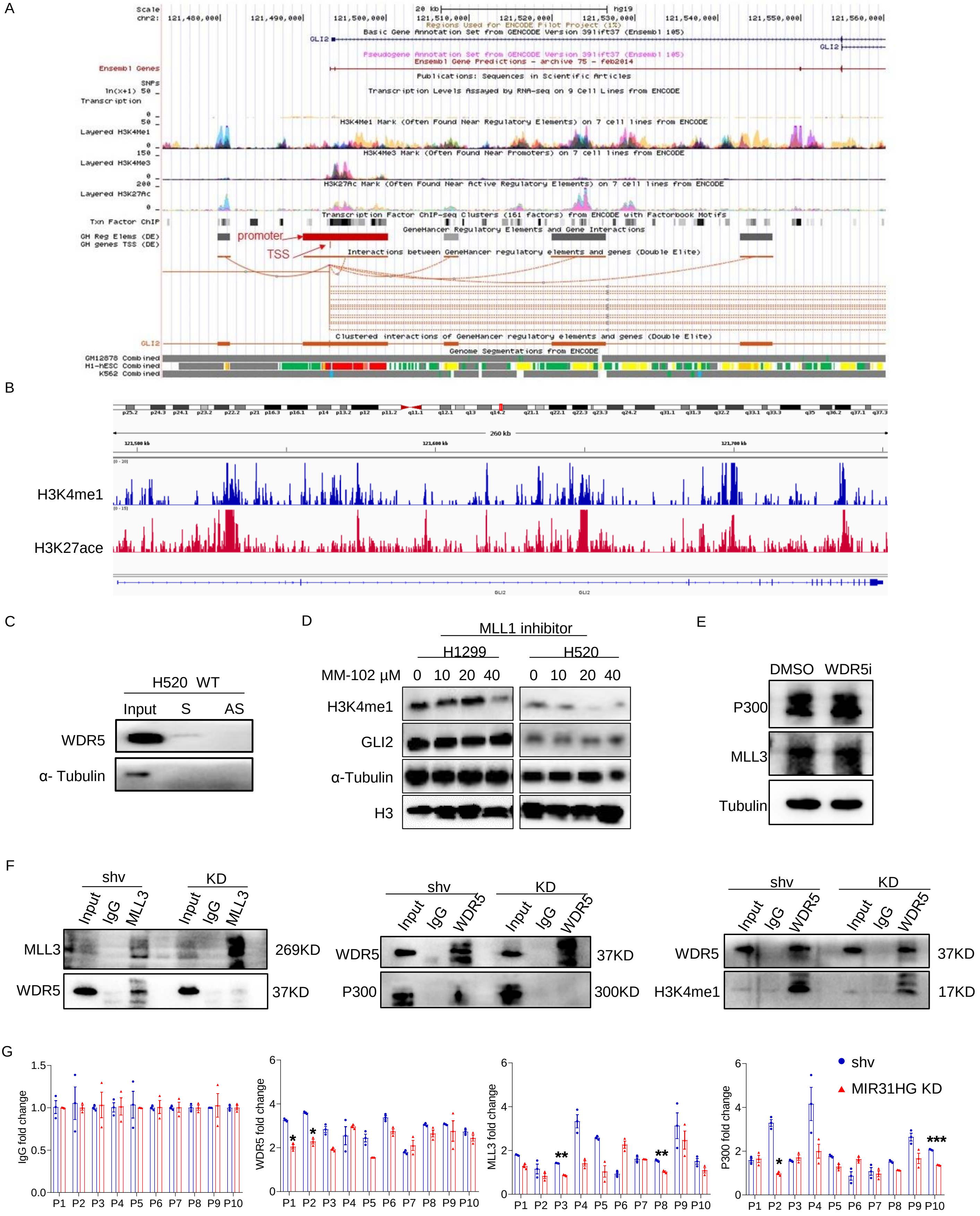

A
Scale
chr2:
hg19
20 kb
121,480,000
121,490,000
121,500,000
121,510,000
121,520,000
121,530,000
121,540,000
121,550,000
121,560,000
Regions Used for ENCODE Pilot Project (1%)
Basic Gene Annotation Set from GENCODE Version 39lift37 (Ensembl 105)
GLI2
Pseudogene Annotation Set from GENCODE Version 39lift37 (Ensembl 105)
Ensembl Gene Predictions - archive 75 - feb2014
Ensembl Genes
Publications: Sequences in Scientific Articles
SNPs
Transcription Levels Assayed by RNA-seq on 9 Cell Lines from ENCODE
Transcription
H3K4Me1 Mark (Often Found Near Regulatory Elements) on 7 cell lines from ENCODE
Layered H3K4Me1
H3K4Me3 Mark (Often Found Near Promoters) on 7 cell lines from ENCODE
Layered H3K4Me3
H3K27Ac Mark (Often Found Near Active Regulatory Elements) on 7 cell lines from ENCODE
Layered H3K27Ac
Transcription Factor ChIP-seq Clusters (161 factors) from ENCODE with Factorbook Motifs
Txn Factor ChIP
GeneHancer Regulatory Elements and Gene Interactions
GH Reg Elems (DE)
GH genes TSS (DE)
promoter
TSS
Interactions between GeneHancer regulatory elements and genes (Double Elite)
Clustered interactions of GeneHancer regulatory elements and genes (Double Elite)
Genome Segmentations from ENCODE
GM12878 Combined
H1-hESC Combined
K562 Combined
B
260 kb
121,500 kb
121,600 kb
121,700 kb
H3K4me1
H3K27ace
GLI2
C
H520 WT
Input
S
AS
WDR5
α- Tubulin
D
MLL1 inhibitor
H1299
H520
MM-102 µM
0 10 20 40
0 10 20 40
H3K4me1
GLI2
α-Tubulin
H3
E
DMSO
WDR5i
P300
MLL3
Tubulin
F
shv
KD
Input
IgG
MLL3
269KD
WDR5
37KD
Input
IgG
WDR5
37KD
P300
300KD
Input
IgG
WDR5
37KD
H3K4me1
17KD
G
IgG fold change
WDR5 fold change
MLL3 fold change
P300 fold change
P1 P2 P3 P4 P5 P6 P7 P8 P9 P10
shv
MIR31HG KD

Fig. S5 H3K4me and H3K27ace landscape in the regulatory region of GLI2 and the effect of MIR31HG on the stability of WDR5/MLL3/P300 complex. A Bioinformatic prediction of H3K4me and H3K27Ace on GLI2 regulatory element generated by USCS genome browser. USCS: University of California Santa Cruz. **B** CUT&Tag sequencing of GLI2 regulatory region in H1299 cells precipitated by H3K4me1 or H3K27ace. **C** WDR5 antibody coprecipitated with biotin-sense-MIR31HG or biotin-antisense-MIR31HG by RNA pull-down assay in H520 cells. Whole cell lysate was labeled as the input and used as the positive control. **D** Protein levels of H3K4me1 and GLI2 in NSCLC cells treated with MLL1 inhibitor detected by immunoblot. **E** The expression of P300 and MLL3 were detected by immunoblot in H520 cells treated with WDR5 inhibitor. DMSO: solvent of WDR5 inhibitor; WDR5i: WDR5 inhibitor applied at 20 μM. **F** The interactions between WDR5/MLL3, MLL3/P300, and WDR5/H3K4me1 were detected in control and MIR31HG knock down group detected by co-IP in H520 cells. shv: pLV backbone; KD: pLV-MIR31HG shRNA #1. **G** Genome precipitated by WDR5, MLL3 and P300 antibodies zoomed at the GLI2 enhancer area by chromatin Immunoprecipitation, respectively in shv or MIR31HG KD NSCLCs. Data are presented as mean ± SEM; significance abundance: * $p < 0.05$, ** $p < 0.01$, *** $p < 0.001$.

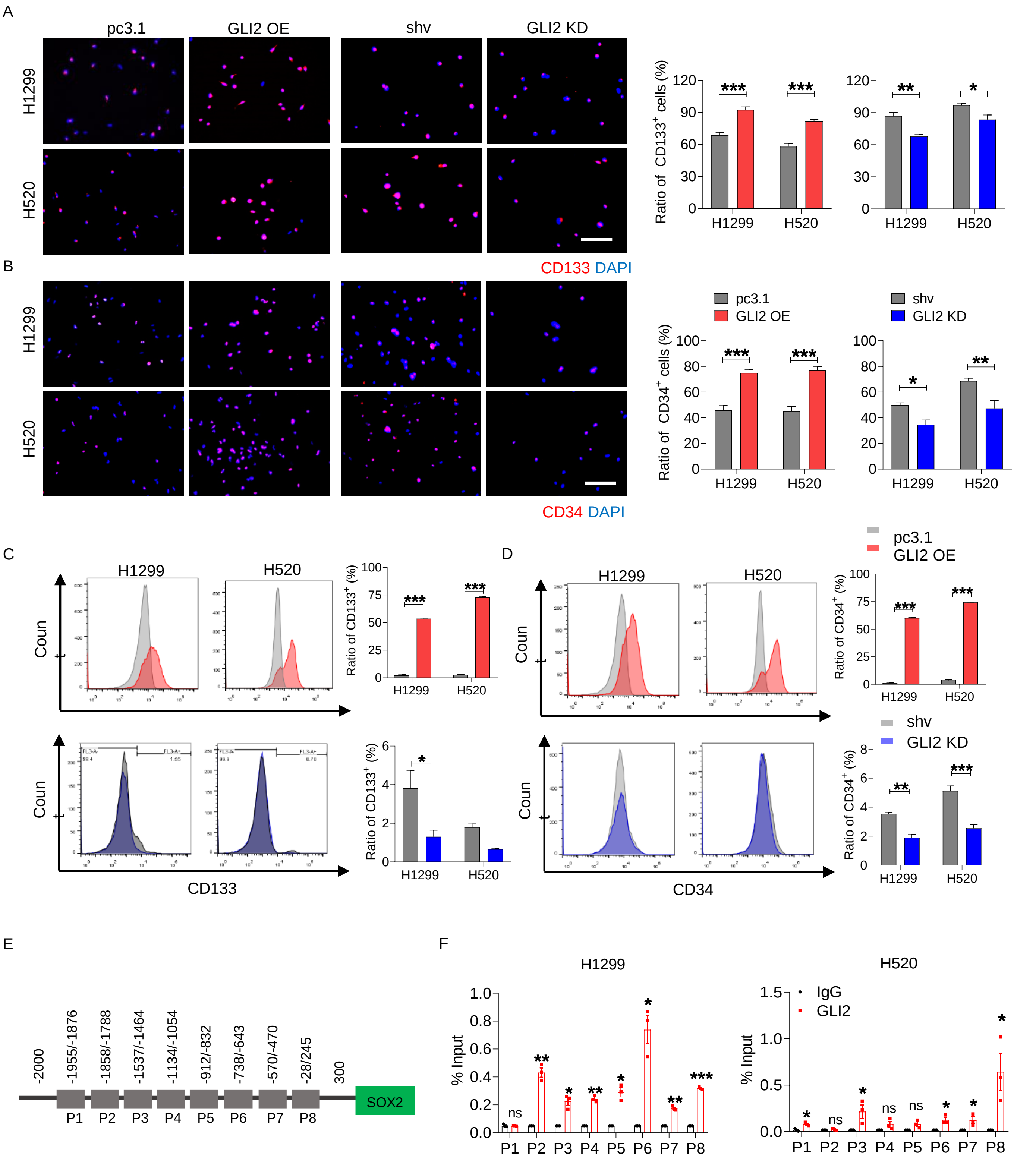

Fig. S6 GLI2 promotes stemness features of NSCLC cells. A, B Immunofluorescent staining of H1299 and H520 cells upon forced GLI2 overexpression or repression, compared to pc3.1 and shv empty vector separately. **C, D** The expression of cancer stemness markers CD133 and CD34 were detected by FACS analysis upon GLI2 overexpression or knockdown compared to levels in empty vector. **E** Schematic map of the primer locations covering promoter of SOX2. **F** Interaction between GLI2 and SOX2 promoter regions (-2000 to +300 around transcriptional starting site) in NSCLC cells explored by chromatin Immunoprecipitation RT-qPCR. Data are presented as mean ± SEM; significance abundance: * $p < 0.05$, ** $p < 0.01$, *** $p < 0.001$.

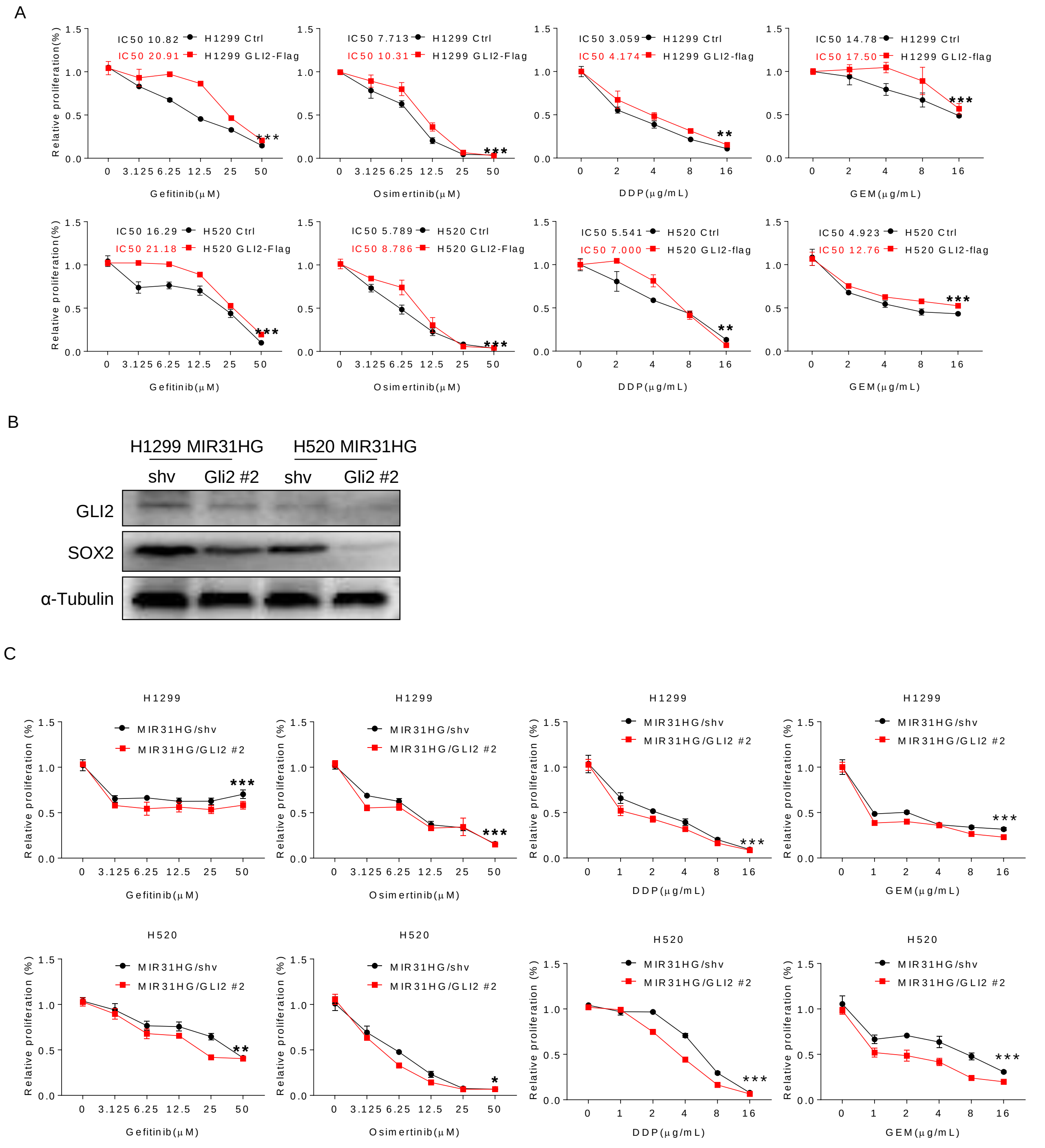

Fig. S7 Repression of GLI2 retards stemness features of NSCLC cells caused by MIR31HG OE. A Growth curve of NSCLC cells treated with gefitinib, osimertinib, DDP, or GEM under GLI2 overexpression, as detected by the CCK8 assay. **B** Protein level of GLI2 and SOX2 upon MIR31HG overexpressing and GLI2 knockdown double transfection measured by immunoblot. **C** Growth curve of NSCLC cells treated by DDP, GEM, gefitinib, or osimertinib, upon MIR31HG overexpressing and GLI2 genetic inhibition. Data are presented as mean ± SEM; significance abundance: * $p < 0.05$, ** $p < 0.01$, *** $p < 0.001$.

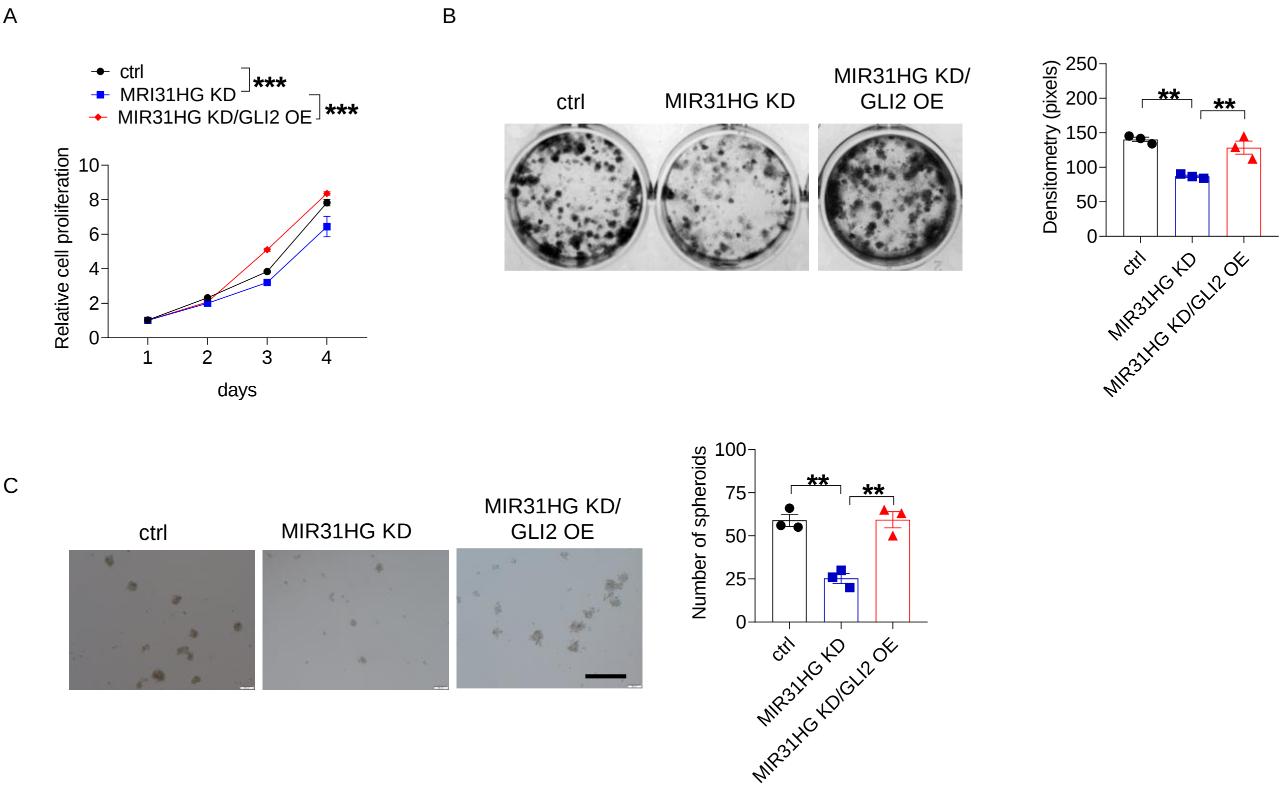

Fig. S8 Overexpression of GLI2 reverses the effect of 31HG knockdown in H520 cells. A Growth curve of MIR31HG knockdown, MIR31HG knockdown and GLI2 overexpression double transfection by the CCK8 assay. **B** Clonal formation assay (left) and quantification (right) of MIR31HG knockdown, MIR31HG knockdown and GLI2 overexpression double transfection. **C** Spheroid formation assay (left) and quantification (right) of MIR31HG overexpression and MIR31HG knockdown double transfection. Scale bar, 200 μm. Data are presented as mean ± SEM; significance abundance: * $p < 0.05$, ** $p < 0.01$, *** $p < 0.001$.

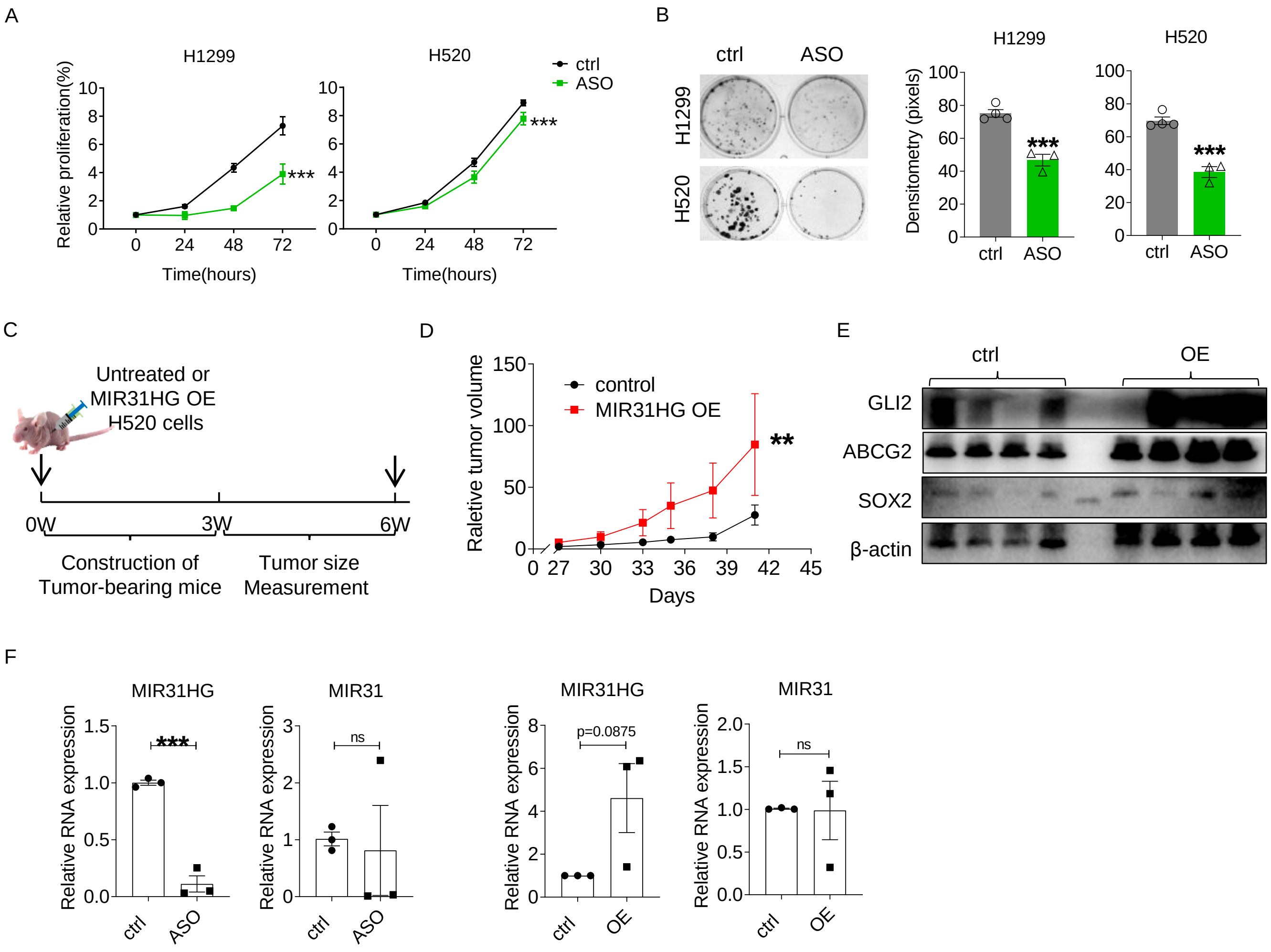

Fig. S9 Antisense oligonucleotide drugs targeting MIR31HG attenuated NSCLCs tumorigenesis both *in vitro* and *in vivo*. A Proliferative capability of lung cancer cells treated by antisense oligonucleotide drugs targeting MIR31HG *in vitro*. Ctrl: control oligonucleotide sequence; ASO: antisense oligonucleotide against MIR31HG. **B** Clonal formation (left) and histogram quantification (right) of lung cancer cells treated by ASO drugs targeting MIR31HG *in vitro*. **C** Schematic illustration of mice experiments. **D** Growth curve of tumor bulk following H520 transplantation between control and MIR31HG OE groups. Control: H520 transfected with pcDNA3.1 backbone; MIR31HG: H520 transfected with pcDNA3.1-MIR31HG. **E** Protein levels of GLI2, ABCG2, and SOX2 in tumor bulk from different groups. **F** The mRNA levels of MIR31HG, GLI2 and MIR31 in tumor bulk from different groups. Data are presented as mean ± SEM; significance abundance: * $p < 0.05$, ** $p < 0.01$, *** $p < 0.001$.

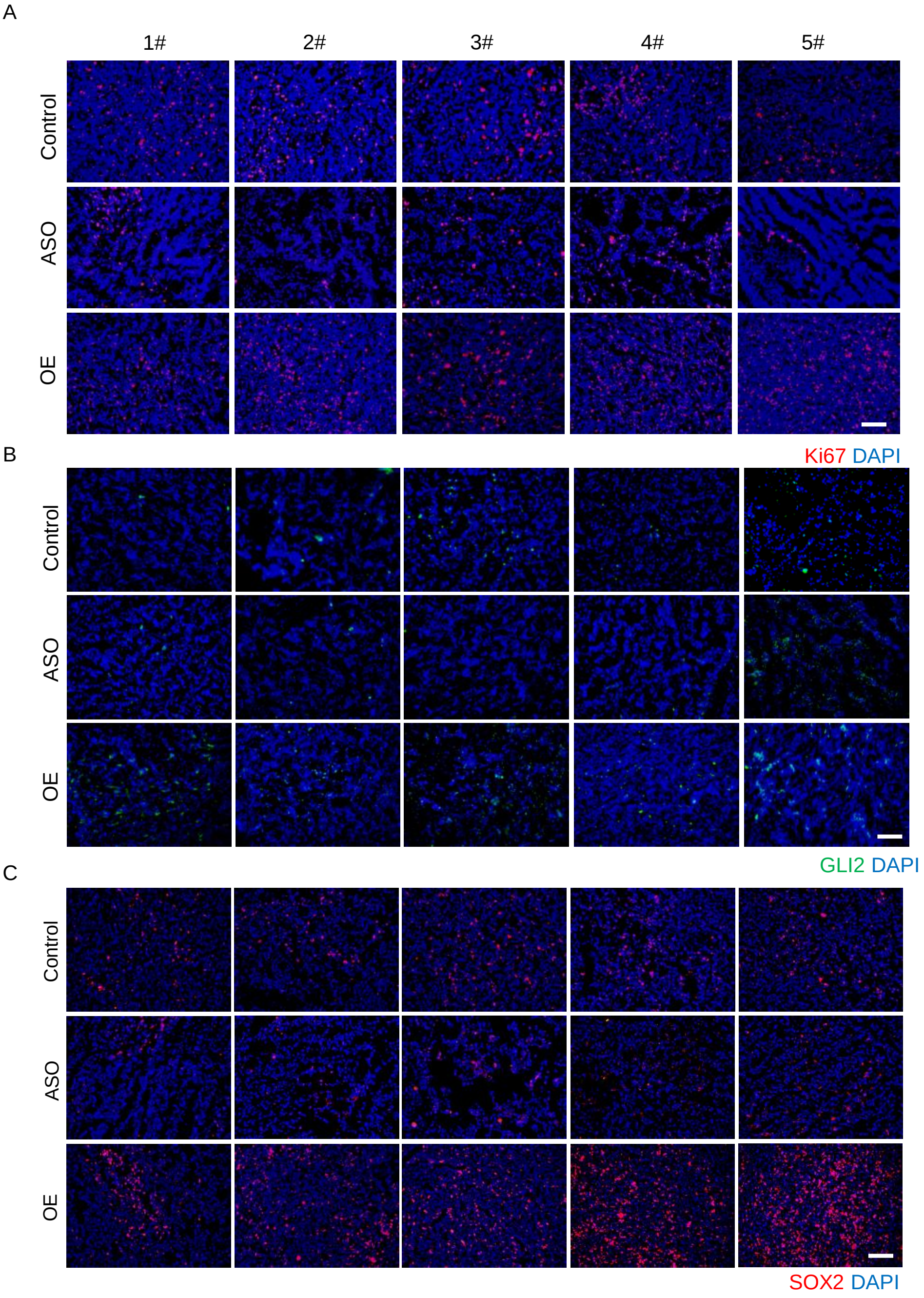

Fig. S10 MIR31HG was positively correlated with GLI2/SOX2 axis in tumor tissues. A, B, C Immunofluorescent staining of Ki67, GLI2 and SOX2, respectively in tumor bulk of different groups. Scale bar, 100 µm. Data are presented as mean ± SEM; significance abundance: * $p < 0.05$, ** $p < 0.01$, *** $p < 0.001$.

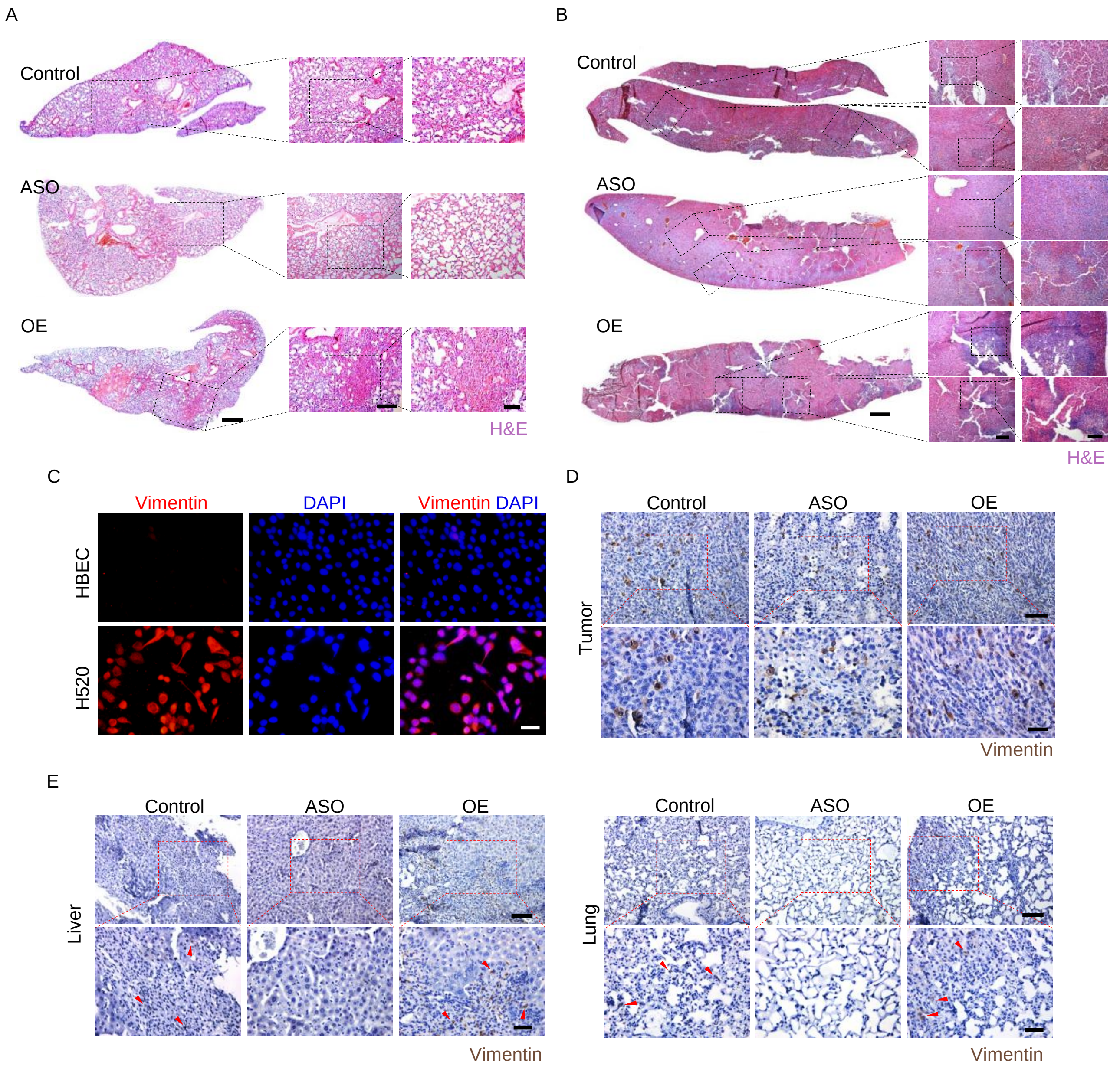

Fig. S11 Tumorigenesis and distal invasion of H520 cells was validated by expression of vimentin. A Hematoxylin-eosin staining of tumor invasion in lung. Scale bars: 500 μm (Left), 200 μm (Mid) or 100 μm (Right). **B** Hematoxylin-eosin staining of tumor invasion in liver. Scale bars: 500 μm (Left), 200 μm (Mid) or 100 μm (Right). **C** Expression of vimentin in H520 cells compared to HBEC cells as negative control detected by immunofluorescent staining. DAPI was used to indicate nucleus. Scale bar, 40 μm. **D** Immunohistochemistry staining of vimentin in tumor bulk from different groups. Scale bars: 100 μm or 40 μm (magnified images). **E** Immunohistochemistry staining of vimentin in metastatic loci from liver and lung specimens. Scale bars: 100 μm or 40 μm (magnified images). Data are presented as mean ± SEM; significance abundance: * $p < 0.05$, ** $p < 0.01$, *** $p < 0.001$.
